# A Stiff Extracellular Matrix Favors the Mechanical Cell Competition that Leads to Extrusion of Bacterially-Infected Epithelial Cells

**DOI:** 10.3389/fcell.2022.912318

**Published:** 2022-06-22

**Authors:** Raúl Aparicio-Yuste, Marie Muenkel, Andrew G. Clark, María J. Gómez-Benito, Effie E. Bastounis

**Affiliations:** ^1^ Department of Mechanical Engineering, Multiscale in Mechanical and Biological Engineering (M2BE), Instituto de Investigación en Ingeniería de Aragón (I3A), University of Zaragoza, Zaragoza, Spain; ^2^ Interfaculty Institute of Microbiology and Infection Medicine, Cluster of Excellence “Controlling Microbes to Fight Infections” (CMFI, EXC 2124), University of Tübingen, Tübingen, Germany; ^3^ Institute of Cell Biology and Immunology/Stuttgart Research Center Systems Biology, University of Stuttgart, Stuttgart, Germany; ^4^ Center for Personalized Medicine, University of Tübingen, Tübingen, Germany

**Keywords:** finite element analysis, cell competition, cell mechanics, traction and monolayer stresses, infection, *Listeria monocytogenes*, epithelial cells, cell extrusion

## Abstract

Cell competition refers to the mechanism whereby less fit cells (“losers”) are sensed and eliminated by more fit neighboring cells (“winners”) and arises during many processes including intracellular bacterial infection. Extracellular matrix (ECM) stiffness can regulate important cellular functions, such as motility, by modulating the physical forces that cells transduce and could thus modulate the output of cellular competitions. Herein, we employ a computational model to investigate the previously overlooked role of ECM stiffness in modulating the forceful extrusion of infected “loser” cells by uninfected “winner” cells. We find that increasing ECM stiffness promotes the collective squeezing and subsequent extrusion of infected cells due to differential cell displacements and cellular force generation. Moreover, we discover that an increase in the ratio of uninfected to infected cell stiffness as well as a smaller infection focus size, independently promote squeezing of infected cells, and this phenomenon is more prominent on stiffer compared to softer matrices. Our experimental findings validate the computational predictions by demonstrating increased collective cell extrusion on stiff matrices and glass as opposed to softer matrices, which is associated with decreased bacterial spread in the basal cell monolayer *in vitro*. Collectively, our results suggest that ECM stiffness plays a major role in modulating the competition between infected and uninfected cells, with stiffer matrices promoting this battle through differential modulation of cell mechanics between the two cell populations.

## 1 Introduction

Cell competition refers to the process whereby less fit cells, often denoted “losers”, are sensed and eliminated by more fit neighboring cells, accordingly referred to as “winners” (Gradeci et al., 2021). This competition between losers and winners is essential for tissue homeostasis but it also emerges during tissue development and can play a role in various pathologies including tumor development (Meyer et al., 2014; Moreno et al., 2019). Although the chemical signals driving the battle between two different cell populations are relatively more explored, an increasing number of studies showcases that mechanical signals such as differential sensitivity to compression during cell crowding are also crucial in driving such interactions (Gradeci et al., 2019; Matamoro-Vidal and Levayer, 2019; Moreno et al., 2019; Bastounis et al., 2021b).

We recently showed that a mechanical competition during late infection drives the collective onslaught and elimination of infected cells out of the epithelial monolayer (Bastounis et al., 2021b). When epithelial cells in monolayer get exposed to low dosage of *Listeria monocytogenes* (*L.m.*), a food-borne facultative intracellular bacterial pathogen, some sparse cells in the monolayer get infected. Within several hours *L.m.* has the ability to replicate intracellularly and spread intercellularly to larger domains containing hundreds of cells. However, at later times post-infection (∼16 h post-infection, hpi), we discovered that surrounding uninfected cells (“winners”) responding to innate immune signals, migrate actively and in a coordinated fashion towards the infection focus, squeezing the infected cells and eventually forcing their massive extrusion (mounding) out of the monolayer (∼24 hpi). Infected cells ultimately die, possibly due to their forceful separation out of their basement membrane, thus suggesting that the “infection mounding” process is a beneficial for the host process in that it obstructs infection dissemination through the cell monolayer. Interestingly, this competition that leads to infected cell elimination is mechanical in nature, and depends on: 1) a decrease in contractility (i.e., traction stresses exerted by the cells on their matrix) of infected as opposed to uninfected neighbors; 2) a lowering in the passive stiffness of infected as opposed to uninfected neighbors, and 3) the presence of cell-cell adhesions since lack of those completely stalls mound formation. Thus, it appears that cell-matrix and cell-cell force transduction as well as cellular stiffness are crucial determinants in driving the mechanical competition that emerges during infection (Bastounis et al., 2022).

Studies conducted over the last decades have underlined the importance of extracellular matrix (ECM) stiffness in regulating important cellular functions such as cell motility, by modulating the cellular traction forces, the intercellular forces and/or the bulk stiffness of cells (Solon et al., 2007; Califano and Reinhart-King, 2010; Borau et al., 2011; Bastounis et al., 2019; Doss et al., 2020). In many different cell types, as ECM stiffness increases, cellular traction force generation also increases, and this effect is particularly prominent in single cells and to a lesser degree in cellular monolayers (Lampi et al., 2016; Zhao et al., 2018; Bastounis et al., 2019). Given that ECM stiffness increases in certain pathologies including fibrotic diseases, cancer and inflammatory bowel diseases (IBD) (Lampi and Reinhart-King, 2018; Onfroy-Roy et al., 2020), it remains still an open question whether varying ECM stiffness would promote or limit infected cell extrusion and, if so, what would be the physical and molecular mechanisms involved. Interestingly, Gradeci et al., showed that changes in the ratio of winner-to-loser cell stiffness altered the kinetics of cell competition between wild-type MDCK cells and cells depleted for the polarity protein scribble, although ECM stiffness was not addressed in this study (Gradeci et al., 2021). Another study on competition between wild-type cells and oncogenically-transformed ones did show that increasing ECM stiffness attenuates extrusion of transformed cells by tuning the dynamic localization of filamin, an important F-actin crosslinking protein (Pothapragada et al., 2022). Thus, it remains unclear whether there are generalizable mechanisms that could predict how ECM stiffness impacts cell behavior and thus the outcome of a cell competition, and whether those would apply to epithelial cells that are infected with intracellular bacterial pathogens, which can replicate intracellularly and also dynamically spread from cell to cell.

To explore such questions, studies often rely on *in vitro* experiments. Such experiments have provided great insight into how intracellular bacteria efficiently spread through epithelial cells in monolayer and on the physical cellular processes that bacteria often hijack to promote their dissemination (Lamason et al., 2016; Faralla et al., 2018). However, *in silico* cellular models and simulations can complement *in vitro* experiments and even facilitate the design of future *in vitro* experiments (Brodland, 2015; Gradeci et al., 2021). Such models present several advantages such as that they are controllable, time-efficient, and cost-effective. Moreover, one can tweak one parameter at a time, thus making it easier to reach causal conclusions. Nevertheless, they do need *in vitro* and *in vivo* models to be properly validated and calibrated. Most infection computational models have focused on the dynamics of bacterial spread in colonies considering contact forces, bacterial growth or the interaction between bacteria and biomaterials (Delarue et al., 2016). Others have focused on the dynamics of intracellular bacterial spread, with the bacteria modeled as particles within two-dimensional (2-D) rigid host cells (Ortega et al., 2019). Recently, we developed a computational, three-dimensional (3-D) finite element model (FEM) to explore the physical mechanisms that drive the squeezing and extrusion of bacterially infected cells during their competition with surrounding uninfected cells (Bastounis et al., 2021b). This simplified model not only validated our experimental results but also predicted that cell-cell adhesions between infected cells and immediate surrounders are necessary for mound formation, a result that we then confirmed experimentally.

Herein, we introduce an extension to our infection computational model with the aim to investigate the previously overlooked role of ECM stiffness in potentially modulating the forceful extrusion of infected cells by uninfected surrounders (Bastounis et al., 2021b). The parameters of our model are selected based on our experimental observations. We find that increasing ECM stiffness promotes the collective squeezing and subsequent extrusion of infected cells due to differential cellular displacements and cellular force generation. Moreover, we discover that an increase in the ratio of uninfected to infected cell stiffness as well as a smaller infection focus size, both promote squeezing of infected cells, and this phenomenon is more prominent on stiffer as opposed to softer matrices. Our experimental findings validate the computational predictions by demonstrating increased collective cell extrusion on stiff matrices and glass as opposed to softer matrices, accompanied by decreased bacterial spread in the basal cell monolayer *in vitro*. Collectively, our results suggest that ECM stiffness plays a major role in modulating the competition between “winner” uninfected cells and “loser” infected cells with stiffer matrices promoting this battle through differential modulation of cell mechanics between the two cell populations.

## 2 Materials and Methods

### 2.1 *In vitro* Experiments

#### 2.1.1 Cell Culture

Epithelial cells type II MDCK cells and type II MDCK cells that express E-cadherin-RFP were a generous gift of the Nelson lab, Stanford University (Perez et al., 2008). MDCK cells were cultured in high glucose DMEM medium (Thermofisher; 10741574) containing 4.5 g/L glucose and supplemented with 10% fetal bovine serum (Thermofisher, 10270106), further referred to as DMEM. They were kept at a temperature of 37°C with 5% CO_2_.

#### 2.1.2 Bacterial Infections and Fixation of Samples for Imaging Mound Volumes

Infection of MDCK cells with *L.m.* was performed as described previously (Bastounis et al., 2021a) using *L.m.* strain JAT607 (Species: *L.m.* 1043S, Genotype/Description: ActAp::mTagRFP) (Ortega et al., 2017). JAT607 *L.m.* express mtagRFP under the control of the ActA promoter which makes them fluoresce only few hours (approximately 4 h) after host cell internalization. The infection assays were performed as follows. Three days prior to infection frozen glycerol stocks of JAT607 were streaked on BHI agar plates containing 7.5 μg/ml chloramphenicol and 0.2 mg/ml streptomycin and incubated at 37°C for approximately 1 day until colonies formed. 16 h prior to infection a 2 ml BHI solution supplemented with 7.5 μg/ml chloramphenicol and 0.2 mg/ml streptomycin was inoculated with JAT607 bacteria from the BHI agar plates and incubated for approximately 16 h in the dark, without shaking at room temperature (RT). The optical density (OD_600_) of the overnight culture was then measured (approximately 0.4), the bacterial cultures were centrifuged at 2000 g for 5 min at RT and re-suspended in 2 ml PBS. 0.5 ml of this bacterial suspension was added to 24 ml DMEM medium. MDCK cells were washed once with PBS and 1 ml of the bacterial-containing DMEM solution was added to each well, so that the resulting multiplicity of infection (MOI) was approximately ∼ 250 bacteria/cell. After 30 min incubation at 37°C with 5% CO_2_ the bacterial-containing medium was exchanged with DMEM containing 5 μg/ml Hoechst (Fisher, 11534886) to stain the host cell nuclei. After 10 min incubation under the previous conditions MDCK cells were washed with PBS three times and DMEM containing 20 μg/ml gentamycin (Fisher, 15820243) was added. 24 h post-infection MDCK cells were washed once again with PBS after which 4% methanol free paraformaldehyde (Thermofisher, 28906) in PBS was added in each well for 10 min. Samples were then washed once in PBS and stored in PBS at 4°C until microscopy imaging was performed.

#### 2.1.3 Mound Volume Calculations, Infection Focus Area and Total Bacterial Fluorescence

Volumes of infected extruded cell domains were calculated using custom MATLAB scripts as previously explained in detail (Bastounis et al., 2021a). Briefly, images of Hoechst-stained MDCK nuclei and of the bacterial fluorescence in and around a given infection focus were taken using a z-spacing of 0.2 μm. For imaging, we used a Zeiss AxioObserver SD Spinning Disk microscope, equipped with an Axiocam 503 mono CCD camera and a Plan-Apochromat 40x/1.4 Oil DIC objective. For imaging the Hoechst-stained host cell nuclei, we used the blue channel (excitation laser 405 nm, emission filter 450/50 nm) and for the mtagRFP-expressing bacteria, we used the red channel: (excitation laser 561 nm, emission filter 600/50 nm). Nuclei located at the basal cell monolayer were disregarded, and only z-stacks of nuclei of cells extruded from that monolayer were considered. We calculated the area occupied by cells in each z-stack slice individually and then determined the total volume of the extruded area using this information. As a proxy of space occupied by cells, we used the signal of the nuclei and applied an alpha shape for area calculations (MATLAB (MathWorks) function alphaShape). First, the individual z-stack images were flatfield and background corrected and then a multi-threshold (between 5 and 3 thresholds per image) was applied to create a binary mask of the nuclei. The area occupied by cells in each z-stack was then multiplied by the height between z-stack slices, in order to determine the volume of the entire extruded domain. The codes used are written in Matlab (Mathworks) and can be found at https://github.com/ebastoun/Infection_mound_volume. For calculating the size of infection foci, we used epifluorescence imaging, and specifically an inverted Nikon Eclipse Ti2 with an EMCCD camera (Andor Technologies) and a 40 × 0.60 NA Plan Fluor air objective. The system was controlled by the MicroManager software. To characterize the efficiency of *L.m.* spread from cell-to-cell through the MDCK cell monolayer, we measured the size of infection foci as previously described (Ortega et al., 2019). The codes used are written in Matlab (Mathworks) and can be found at https://github.com/Fabianjr90/Listeria_focus_shape_analysis.

#### 2.1.4 Fabrication of Polyacrylamide Hydrogels

Polyacrylamide hydrogels were prepared on glass bottom 24-well plates (MatTek, P24G-1.5-13-F) as previously described in (Bastounis et al., 2021a). Briefly, glass coverslips were pretreated with 0.5 M NaOH for 30 min at RT, rinsed with water and then incubated for 5 min at RT with 2% APTS ((3-Aminopropyl)triethoxysilane, Sigma, A3648-100ML) in 95% EtOH. After a rinsing step with water, the coverslips were incubated for another 30 min with 0.5% Glutaraldehyde (Sigma, G6257-100ML), rinsed again with water and finally dried at RT. Polyacrylamide hydrogels were built in two layers to achieve a sufficiently thin layer of fluorescent beads on the surface. 3 kPa hydrogels were prepared by mixing 5% acrylamide (Sigma, A4058-100ML) and 0.1% bis-acrylamide (Fisher, 10193523). 35 kPa hydrogels were prepared by mixing 8% acrylamide and 0.26% bis-acrylamide. Polymerization was initiated by addition of 0.06% ammonium persulfate and 0.43% TEMED. The first layer was created by adding 3.6 μL of the acrylamide mix on the glass coverslip and covering it with a 12 mm round cover glass which was gently pressed on top to create a flat surface. During the polymerization of the first layer, an additional 0.03% of 0.2 μm fluorescent beads (Thermofisher, F8810) was added to the second layer solution. 2.4 μL of the second layer solution were then added on top of the first layer after removing the glass coverslip. A 12 mm round cover glass was again placed on top to generate a flat polyacrylamide layer containing tracer beads. After polymerization and removal of the round cover glass the gels maintained in 50 mM Hepes, pH 7.5 were UV sterilized for 1 h. Gels were then activated by addition of 200 μL of 0.5% w/v heterobifunctional cross-linker Sulfo-SANPAH (Fisher; 10474005) in 1% dimethyl sulfoxide (DMSO, Sigma, D2650-5X10ML) in 50 mM HEPES, pH 7.5, and exposure to UV light (*λ* = 302 nm) for 10 min. The Sulfo-SANPAH was then removed, and the gels were washed with 50 mM HEPES, pH 7.5. Gels were incubated overnight with 200 μL of 0.25 mg/ml rat tail collagen (Thermofisher, A1048301) and the following day washed once with 50 mM HEPES, pH 7.5 and stored in the same buffer.

#### 2.1.5 Traction Force Microscopy (TFM)

TFM was performed as previously described (Lamason et al., 2016; Bastounis et al., 2021a). More specifically, polyacrylamide hydrogels were equilibrated for 30 min at 37°C with cell media prior to cell seeding. Subsequently, 4 × 10^5^ MDCK cells were seeded on the hydrogels. 24 h post-seeding, cells were stained with 5 μg/ml Hoechst stain (Fisher, 11534886) for 10 min. Cells were washed once in PBS, and 1 ml of Leibovitz’s L-15 Medium, without phenol red (Fisher, 21083027) and supplemented with 10% fetal bovine serum was added in each well. The multi-well plate was then transferred to the microscope stage for initiation of time-lapse acquisition. For imaging we used an inverted Nikon Eclipse Ti2-E with a Prime BSI sCMOS camera (Teledyne Photometrics) using a ×40 CFI Super Plan Fluor ADM ELWD objective with a NA 0.60 and the NIS-Elements Microscope Imaging Software (Nikon Metrology). Multi-channel images of the phase contrast image of cells, the nuclei fluorescence and the tracer beads’ fluorescence were taken every 10 min. After approximately 8 h of imaging, the acquisition was stopped and 10% SDS was added to the wells to detach the cells from their matrix. Reference images of the beads in the undisturbed hydrogel surface were acquired. We used a particle image velocimetry-like technique to compare the image of the tracer beads at each instance of time with the reference image to determine the beads’ displacements in MATLAB (MathWorks) (Gui and Wereley, 2002). We used interrogation windows of 48 × 24 pixels (window size × overlap). Calculations of the two-dimensional traction stresses that MDCK cells in monolayer exert on the hydrogel are described elsewhere (Bastounis et al., 2014; Lamason et al., 2016) and were performed also in MATLAB. For calculation of the number of nuclei in each recording the images of Hoechst-stained nuclei were used and the Fiji (ImageJ) plugin Trackmate was used for segmentation of cell nuclei and tracking. Output data from Trackmate were exported as xml files and read in Matlab to calculate cell speed based on nuclear motion (Hayer et al., 2016).

#### 2.1.6 Monolayer Stress Microscopy (MSM)

Traction stresses were retrieved from the TFM measurements described above. We assumed perfect cell-substrate adhesions and complete confluence of the monolayer. We neglected the components of the traction forces perpendicular to the plane of the monolayer. We considered the traction forces exerted by the cells on their substrate are equal to the forces applied by the substrate on the cells but pointing in the opposite direction (third Newton’s law). We also assumed that the monolayer displayed a uniform and constant thickness, which was much smaller than the dimensions of the monolayer. Thus, this methodology is only valid at the early stages of intercellular bacterial spread, just before mounding occurs (approximately 16 h post-infection). The domain was discretized with a finite element mesh (element size 28.352 μm) and monolayer stresses (that is inter- and intra-cellular stresses) were computed under the hypothesis of plane stress [for more details of the methodology see Tambe et al. (2011); Aparicio-Yuste et al. (2022)]. We initially considered that the stiffness of both uninfected surrounders and infected cells is the same (R_*E*_ = 1) but also ran MSM for the case where infected cells are four-fold softer than uninfected surrounders (R_*E*_ = 4) and confirmed that qualitatively the tangential monolayer stresses did not look significantly different. Custom-written scripts are publicly available at https://github.com/ebastoun/Monolayer-Stress-Microscopy.

### 2.2 *In silico* Experiments

We built an *in silico* model of the epithelial monolayer considering both infected and uninfected surrounder cells as well as cell-cell and cell-ECM adhesion contacts. The monolayer resided on either a 3 kPa, 10 kPa, 20 or 35 kPa ECM or on a 2 GPa glass coverslip.

#### 2.2.1 The Geometry and Material Properties of our Model

Although the ECM microstructural composition and mechanical properties are highly complex and vary at different length scales (Sun, 2021), for simplicity we assumed that the matrix where cells reside is a continuum medium and as such an average value of its mechanical properties was chosen. We also assumed that the matrix is an elastic isotropic material, as proposed previously (Kandemir et al., 2018). Epithelial cells were simulated in this work as individual components. Although the cell monolayer structure and functions arise from individual entities or cells, we assumed that cells in the monolayer act as a collective since every given cell interacts with its neighbors through cell-cell junctions. Cells were simulated as regular hexagonal prisms despite the more diverse topology they show in a monolayer. This hexagonal pattern is the most frequent polygon type in cell monolayers since it maximizes the space filled by cells in a tissue (Lecuit and Lenne, 2007). Each individual cell was divided into three domains: the contractile, the adhesive and the expanding/protrusive (Bastounis et al., 2021b). This enabled us to simplify the cell architecture and allowed us to simulate computationally cell dynamic changes, such as cellular deformations, cell-ECM traction adhesions and cell protrusions. Additionally, both active and passive cell behaviors were considered in our model. The passive was simulated so to account for the stiffness arising from the cellular cytoskeleton (e.g., the microtubules or intermediate filament network), whereas the active accounted for the actomyosin contractile apparatus and actin polymerization (Moreo et al., 2008; Borau et al., 2011).

#### 2.2.2 Cell-ECM and Cell-Cell Mechanical Interactions Considered

The mechanical interactions of cells with their ECM and between each other were taken into account in our model through cell-ECM contact and cell-cell interactions, respectively. Cell-cell interactions were considered through a continuum approach, assuming perfect adhesions through a linear elastic thin element that transmits the loads between cells. Thus, cells were able to sense forces from their neighbors and interact with them. Cell-ECM adhesions were simulated by a cohesive contact interaction between two surfaces, the given cell and the ECM. The higher the stiffness of the contact, the more difficult the relative displacement between the two surfaces. Furthermore, we worked under the assumption of perfect cell-ECM adhesions, meaning that all the force is transmitted from the cell to the ECM and vice versa.

#### 2.2.3 Assumed Mechanotransduction Scheme Used by Non-infected Cells to Detect Infection

To better examine the impact of infection on the collective squeezing that leads to extrusion of infected epithelial cells, we simulated a cellular cycle where certain cells (e.g., infected) are allowed to experience different degrees of protrusion or strength of adhesion to their ECM. In settings not involving infection (uninfected case), all cells presented the same mechanical behavior. To simulate infection, cells infected with bacteria were assumed to have distinct mechanical properties as compared to neighboring uninfected cells (infected case). More specifically and based on previous experimental findings, infected cells were considered softer and with decreased active stiffness as compared to neighboring uninfected cells (Bastounis et al., 2021b).

For simplicity, bacterial infection was considered fixed, that is, bacteria did not spread or replicate intracellularly during the simulation period. For both infected and uninfected monolayers, we analyzed the cellular behavior over the course of this cellular cycle and according to a mechanotransduction scheme. Briefly, all cells were first exposed to a round of contraction in order to sense and bear loads from their neighboring cells and their ECM. This contraction resulted in cell-ECM displacements and allowed the cells to move with respect to their ECM. If the cell-ECM displacements were large when cells contract, we assumed new cell-ECM adhesions would form, followed by a new cycle of contraction. Once new adhesions were formed, cellular protrusion would occur only if the given cell exhibited asymmetry in its tensional distribution (in the direction of the stress gradient). This means that the cell actively responded to the non-symmetrical distribution of the stresses by protruding. In our simulations, this occurred only during infection and was observed only in uninfected cells close to the infection focus. These cells exhibited tensional asymmetry and thus protruded towards the direction of minimum stress, which lied at the edge of the infection focus.

To summarize, we simulated and examined the 3D dynamics of a cell monolayer residing on a flat ECM of a given stiffness in two particular cases: uninfected and infected condition. We considered that in the uninfected case, all cells in the monolayer exhibited similar mechanical properties and interactions with their ECM. However, under conditions involving infection, cell-ECM traction adhesions of infected cells were weakened compared to uninfected surrounder cells, and new strong traction adhesions were formed along surrounding uninfected cells (where the cells sense there was a large relative displacement with respect to the ECM). In turn, the uninfected cells protruded towards the infection focus due to their tensional asymmetry.

#### 2.2.4 Implementation

The mechanical interactions of infected cells with surrounding uninfected cells in a monolayer were analyzed with a three-dimensional, finite element model using the commercial software ABAQUS CAE 6.14 (Dassault systèmes Simulia Corporation). The general model is described elsewhere (Bastounis et al., 2021b). Based on this model, we built here a FEM of infected and uninfected cell monolayers taking into account additional considerations as outlined below. Regarding the geometry, we assumed two main entities: the ECM and the cells. The parameters characterizing the cells and ECM domains are summarized (Table 1) and were chosen based on previous *in vitro* studies (O’Dea and King, 2012; Schmedt et al., 2012; Escribano et al., 2019). Overall, 217 individual cells and their junctions coexisted, collectively forming a hexagonal monolayer. This hexagonal pattern of the monolayer was selected to avoid artifacts arising from asymmetries.

**TABLE 1:** Summary of the features of the computational model.

Domain	Geometry	Dimensions (*μm*)	Mechanical properties
ECM	Rectangular prism	220 × 220 × 20 (LxWxH)	*E* = 3, 10, 20, 35 kPa and 2 GPa (glass); *ν* = 0.45
Cell	Hexagonal prism	Hexagon side length = 7; Height = 7	*E* = 1 kPa (uninfected cells); *ν* = 0.48
Cell-cell junction	Thin sheet	7 × 0.01 × 7 (LxWxH)	*E* = 1 kPa; *ν* = 0

Our model is composed by four main domains: uninfected cells (Ω_*uninf*_), infected cells (Ω_inf_), cell-cell junctions (Ω_*junction*_) and ECM (Ω_*ECM*_). Concerning material properties, cells and ECM were assumed to behave as linear elastic materials (Escribano et al., 2018); as we were not interested in long-term effects, we just simulated a short period of time (one contraction-protrusion cycle). The Young’s modulus (measure of stiffness) of uninfected surrounder cells was considered of the order of 1,000 Pa, while that of infected cells of 250 Pa, as previously measured by Atomic Force Microscopy (AFM) experiments (Bastounis et al., 2021b). Cell-cell junctions were assumed to have a Young’s modulus of 1,000 Pa, and this value was selected so that the continuity of the mechanical properties of the monolayer is preserved. To interrogate the role of ECM stiffness on infected cell squeezing, we considered different ECM stiffnesses in our simulations, namely: soft, stiff and glass matrices with elastic moduli of 3 kPa, 10 kPa, 20 kPa, 35 kPa, and 2 GPa, respectively, values corresponding to the polyacrylamide hydrogels we built for our *in vitro* experiments and the glass coverslips commonly used. The Poisson’s ratios were set to 0.48 (Moreo et al., 2008) and 0.45 (Alvarez-González et al., 2017) for cells and ECM, respectively, since both are nearly incompressible. We modeled the cells’ active and passive behavior through two different meshes in the model with shared nodes. This strategy allowed us to separate active protrusion and contraction from passive contractility.

In order to simulate cell contraction and expansion/protrusion, we followed the analogy of the thermoelastic expansion equations governing volumetric changes in both contraction and expansions processes (Vujošević and Lubarda, 2002; Hervás-Raluy et al., 2019; Nieto et al., 2020). We assumed the contractile part of the cell was exposed to a negative volumetric change, decreasing its volume. On the contrary, the protruding part of the cell was subjected to a positive volumetric change, increasing its volume. According to our experimental observations, minimal change in cell height was observed during contraction, remarking the importance of cell contraction in the plane of the monolayer. Therefore, we assumed non-isotropic contraction, i.e., the contraction occurred just in the plane of the monolayer to avoid changes in cell height. In such manner, we generated area changes in the plane of the monolayer, but not in the normal direction, thus the Poisson’s coefficient of the active part was set to zero and the same was applied to cell-cell junctions (Table 1 and Table 2).

**TABLE 2:** Mechanical parameters in our computational model.

	Uninfected		Infected	
	*E* (Pa)	*ν* (−)	*E* (Pa)	*ν* (−)
Passive	500	0.48	125	0.48
Active	500	0	125	0
Cell	1,000	—	250	—

Cell-cell interactions were simulated in ABAQUS through a contact interaction, where the normal direction of the contact behaved as a “hard” contact and the tangential direction acted as a frictionless surface. However, we used surface-to-surface contacts to model cell-ECM adhesions under the assumption of perfect cell-ECM adhesions. The domain of the cell referred to as adhesion site was attached to the ECM surface via a cohesive contact. The strength of the traction adhesions (active cellular adhesion sites that transmit traction forces to the ECM) was determined by the stiffness of the matrix **K** and its stiffness coefficients (K_*x*_, K_*y*_, K_*z*_), where K_*x*_ and K_*y*_ are the two shear traction adhesions in the plane of the ECM and K_*z*_ the normal traction adhesion to the plane of the ECM. The higher the stiffness coefficients K_*i*_, the stronger the traction adhesion between the cell and the ECM is and the lower the K_*i*_, the weaker the traction adhesion is. By tuning these parameters, we differentiated three types of cell-ECM contacts. The first type of contact described the formation of new adhesions at the edge of the infection focus on the side of the surrounding uninfected cells, with the stiffness coefficients (K_*x*_, K_*y*_, K_*z*_) = (10, 10, 10) kPa. These high values created a strong cell-ECM contact which is one of the mechanisms that uninfected cells use to fight against infected cells, by squeezing them and eventually eliciting their extrusion. The second type of contact is a general cell-ECM contact whose stiffness coefficients were set to (0,0,0.1) kPa. This condition represented a general cell-ECM attachment, meaning that cells could move in the plane but they could not be separated from the ECM. The third contact concerned infected cells which present weaker cell-ECM traction adhesions and therefore, we assumed their stiffness coefficients were (0,0,0.001) kPa. This distinction between infected and uninfected traction adhesion coefficients was not considered in our previous model (Bastounis et al., 2021b).

The ECM was meshed with standard linear hexahedral solid elements, whose mesh density was higher at the center of the upper surface. Both cell-cell junctions, regions of the cell referred to as protrusion and adhesion, were meshed with linear hexahedral solid elements in order to obtain coincident nodes and regular connectivity. The cell contraction part was meshed with linear triangular prisms to keep the continuity of the mesh. Additionally, to account for the passive and active behavior of the cell, we used two superimposed meshes to which we associated the mechanical properties of the active and passive part of the cell (Table 2). The model resulted in 93.948 nodes and 148.239 elements. As boundary conditions, we assumed all displacements were prevented at the bottom surface of the ECM. In addition, the displacements of all the cells that were at the edge of the monolayer were restricted. These conditions were consistent with our experimental setup. Furthermore, our *in vitro* experiments were performed by choosing the field of view to be near the center of the well on which the cellular monolayer was formed, thus edge effects should be negligible.

To provide novel insight into the role of ECM stiffness in promoting infected cell squeezing, we run simulations using our computational model. First, we tested the impact of ECM stiffness in mound formation by assigning distinct values of ECM stiffness. We considered matrices that are soft, stiff and infinitely stiff (i.e., glass) with an elastic modulus of 3 kPa, 10 kPa, 20 kPa, 35 kPa and 2 GPa, respectively. Second, we investigated how infection spread affects mounding by considering different size infection foci comprised by varying number of infected cells on both soft and stiff matrices. Lastly, we examined how the ratio of uninfected to infected cell passive and active stiffness affects mound formation when ECM stiffness varies. We ran several cases within the same range of the ratio of uninfected to infected cell stiffness (R_*E*_), which we defined as:

$$R_{E}=\frac{E_{\mathrm{uninf}}}{E_{\inf}}$$ (1)

where *E*_*uninf*_ the Young’s modulus of uninfected cells and *E*_inf_ the Young’s modulus of infected cells.

## 3 Results

### 3.1 Epithelial Cells Exert Higher Traction Stresses When Residing on Stiff as Compared to Soft Matrices

**FIGURE 1:**
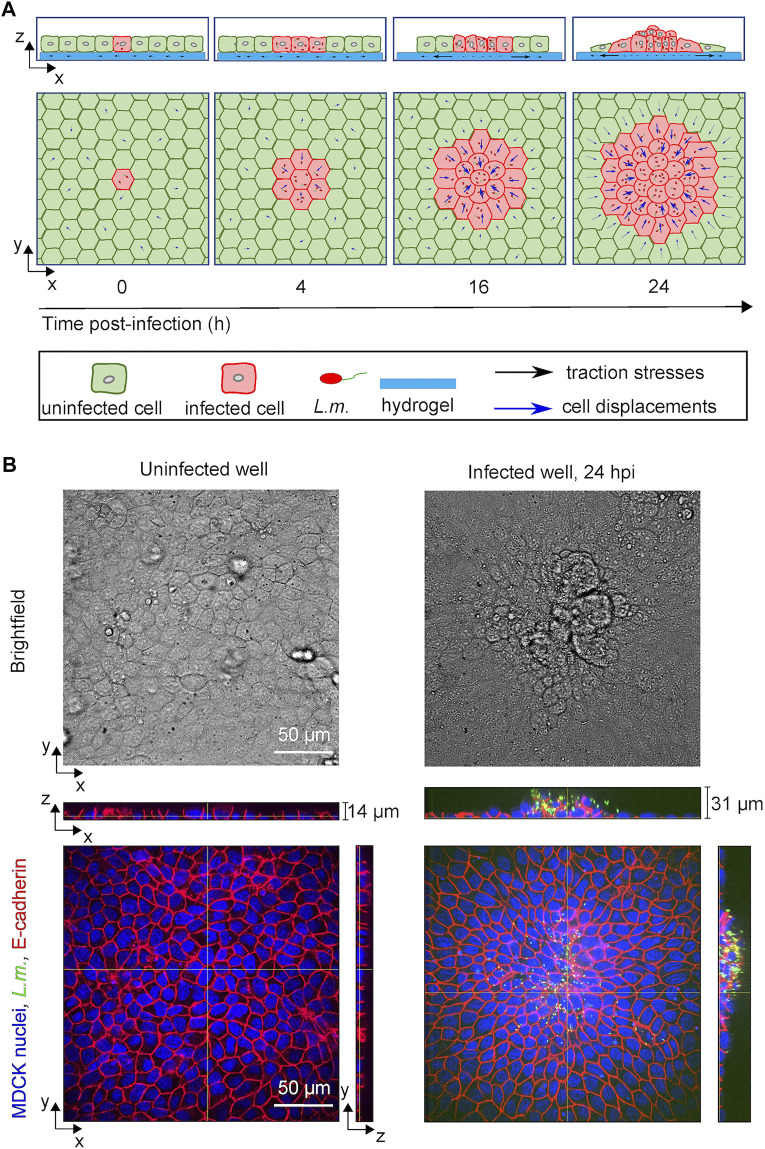
*L.m.*-infected epithelial cells in monolayer get collectively extruded at late infection. **(A)** Schematic illustration showing the time course of *in vitro* infection of host epithelial cells with *L.m.* so that infection mounds emerge at late infection (24 hpi). Initially a single cell gets infected by *L.m.* (0 hpi), but as time proceeds bacteria replicate and spread to neighboring cells (4 hpi). Uninfected surrounder cells (green) start actively migrating towards the infection focus comprised by several infected cells (red), while softer and less contractile infected cells get squeezed (16 hpi) and eventually extruded (24 hpi) out of the cellular monolayer. **(B)** Representative brightfield image (top row) and orthogonal views (bottom row) of *L.m.* fluorescence in green, E-cadherin in red and MDCK nuclei in blue for cells originating from an uninfected well (left column) and from a *L.m.*-infected well with the field of view shown focused around and infection focus at 24 hpi (right column).

**FIGURE 2:**
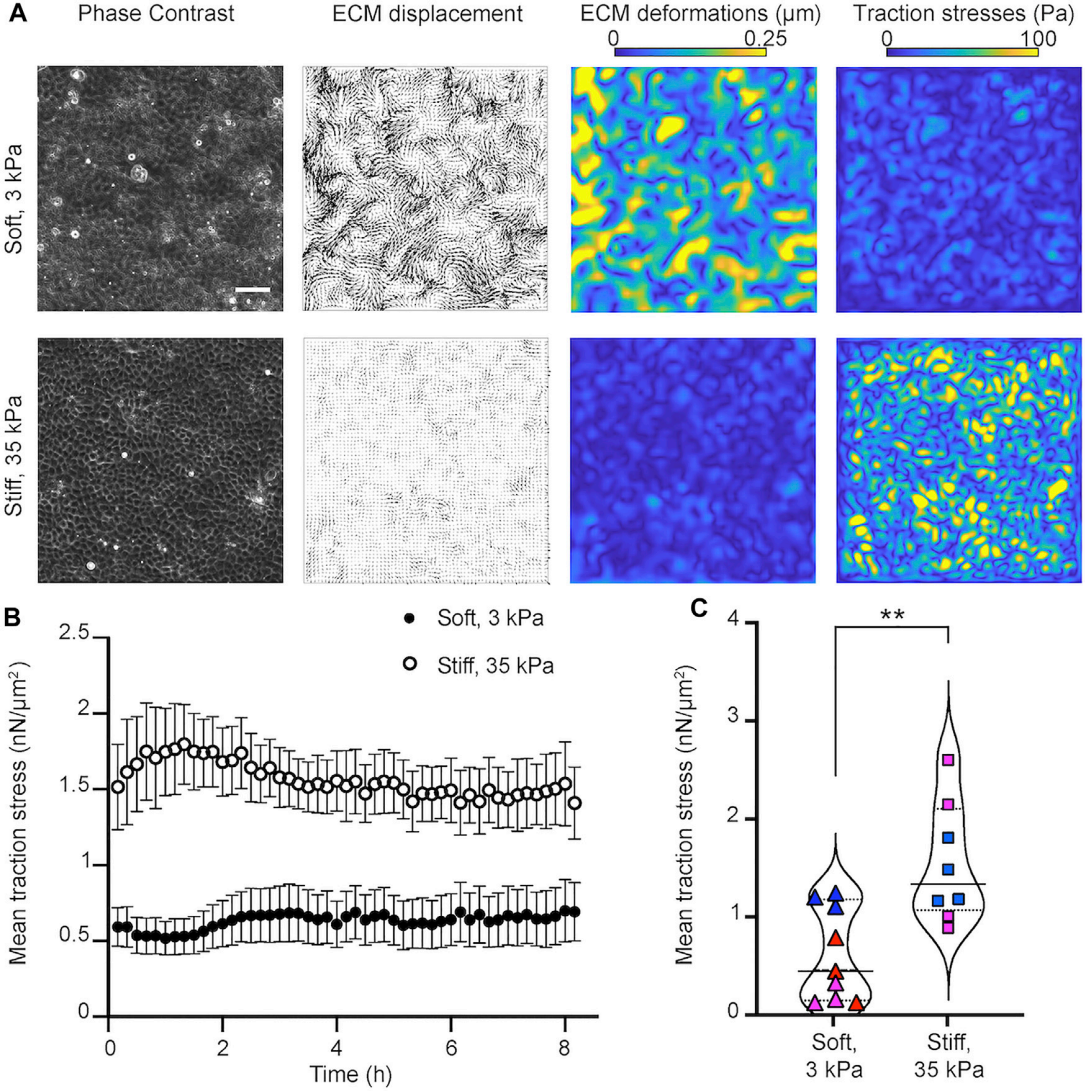
Epithelial cells in monolayer exert higher traction stresses when residing on stiff as opposed to softer matrices. **(A)** Traction force microscopy performed on MDCK cells residing on soft, 3 kPa (upper row) and stiff, 35 kPa (bottom row) hydrogels. Columns of a representative time instance show: phase contrast image of cells, displacements imparted by the cells on the hydrogel (vectors), corresponding deformations (colormap shows magnitude, μm) and traction stresses exerted by cells (colormap shows magnitude, Pa). Scale bar is 50 μm. **(B)** Plot of the mean traction stress magnitude (*y*-axis) versus time (*x*-axis). Mean ± SEM are shown for *N* = 8 and *N* = 9 TFM recordings performed on stiff 35 kPa and soft 3 kPa gels, respectively. **(C)** Violin plots of the mean traction stress magnitude for all recordings and time points shown in panel **(B)**. Symbols show the time average of the mean traction stress for each recording. Same color points correspond to recordings performed the same day on different wells. Solid line indicates the mean and dotted the STD of all time averages. **: p < 0.001, Wilcoxon Ran Sum test.

We have shown that during late infection of epithelial cells with *L.m.* ( > 16 hpi) a mechanical competition takes place leading to squeezing of softer and less contractile infected cells by stiffer and more contractile uninfected surrounder cells, which actively migrate with high speeds towards the infection focus (Bastounis et al., 2021b) (Figure 1A). As a result of this mechanical battle, infected cells are extruded out of the basal monolayer and pile up forming large 3D “mounds” whose height typically exceeds 30 μm, which is at least three-fold higher than the typical height of cells in non-infected cell monolayers (Figure 1B). Given that it is well known that cell mechanics are modulated by ECM stiffness, we hypothesized that ECM stiffness will impact cellular stiffness and traction force generation and that these, in turn, would impact formation of infection mounds. To assess whether ECM stiffness affects the traction stress-generating ability of cells and whether the magnitude of traction stresses is modulated based on ECM stiffness, we cultured Madin-Darby Canine Kidney (MDCK) epithelial cells as confluent monolayers on hydrogels of varying stiffness. We chose this cell line because it forms polarized and homogeneous monolayers in tissue culture and have broadly been used in studies examining *L.m.* infection (Ortega et al., 2019; Bastounis et al., 2021b). Cells were placed on collagen-I coated soft polyacrylamide hydrogels of 3 kPa or stiff hydrogels of 35 kPa. These values were chosen based on previous studies reporting that the elasticity of intestinal ECM broadly ranges from 1 to 68 kPa (Onfroy-Roy et al., 2020). Hydrogels were embedded with tracer beads such that, as cells in the monolayer pull on the hydrogels via their focal adhesions and on each other via cell-cell adhesions, tracer bead movement can be monitored via time-lapse microscopy. Using the fluorescence images of the beads we inferred the deformations that cells imparted on the gels and the traction stresses they exerted on to it through Traction Force Microscopy (TFM) (Figure 2A and Supplementary Movie S1). 3 kPa is the lowest stiffness we examined since as reported, MDCK on low stiffness gels ( < 3 kPa) do not form monolayers but rather aggregate-like structures (Balcioglu et al., 2020). 35 kPa gels are considered as our stiffest condition, since this was found to be the highest stiffness on which the cells can still deform the gels at enough resolution to be measured accurately with TFM (data not shown).

By seeding MDCK cells at a concentration of 4 × 10^5^ cells per well, on wells from a 24-well plate, we found that MDCK were able to form confluent monolayers with similar number of cells on soft 3 kPa and stiff 35 kPa gels 24 h post-seeding (Supplementary Figure S1A). Moreover, at these high cell confluence conditions, the speed of migration of cells was minimal (∼ 0.08 μm/min) and similar on soft and stiff hydrogels (Supplementary Figure S1B). Nevertheless, MDCK residing on stiff 35 kPa gels generated significantly higher traction stresses but imparted reduced deformations as opposed to the softer 3 kPa gels where deformations were higher but traction stresses were overall lower. As expected, the average traction stresses generated by cells pertaining in a field of view were significantly higher for cells residing on stiff as compared to softer matrices (Figures 2B,C). Therefore, we conclude that under conditions that do not involve infection and where epithelial cells form a highly confluent monolayer, that is, at steady state, cells exert higher traction forces on stiff 35 kPa hydrogels as opposed to softer 3 kPa ones.

### 3.2 Considerations and Assumptions of the Infection Computational Model That Accounts for the ECM Stiffness

Our experimental results demonstrate that epithelial cells in monolayer exert higher traction stresses when residing on stiff as opposed to softer matrices. Given this finding, we wondered whether ECM stiffness would similarly impact cell produced traction stresses if we were to use and further develop our model to run *in silico* infection experiments (Bastounis et al., 2021b). Using this model, in turn, we could examine how cellular traction stresses vary as a function of ECM stiffness also when a focus of infected cells is present within the monolayer.

**FIGURE 3:**
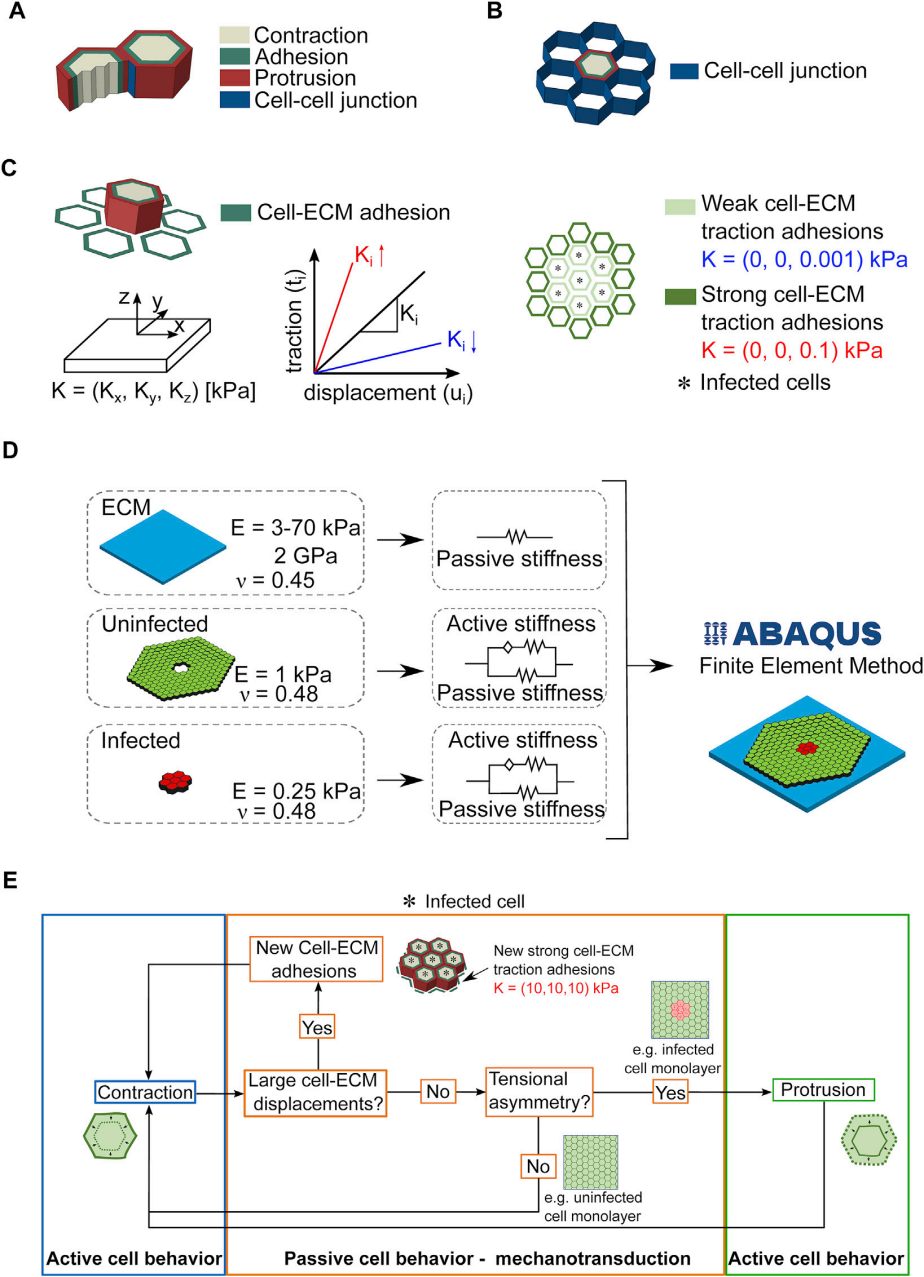
Considerations and assumptions of the infection computational model. **(A,B)**. Sketch showing that cells are approximated in the computational model as hexagonal prisms that form cell-cell junctions with six neighboring cells and are partitioned in three domains: contractile, adhesive and protrusive **(A)**. Cell-cell junctions are modeled as linear thin elastic elements **(B)**. **(C)** Left schematic illustration shows how the cell produced traction stresses exerted to the ECM are modeled through surface-to-surface contacts between the cell and its ECM. The strength of the traction adhesions is characterized by **K** through its stiffness coefficients (K_*x*_, K_*y*_, K_*z*_), with z being the normal direction to the surface, and x and y the directions in the plane of the surface. The higher (lower) the value of K_*i*_ (for i equal to x, y or z), the stronger (weaker) the traction adhesions shown in red (blue). Right schematic illustrates an *in silico* infection focus with infected cells denoted by an asterisk. In our model, unlike surrounding uninfected cells, infected cells form very weak traction adhesions with the ECM. **(D)** Sketch depicting the domains of the Finite Element Model (FEM) used to compute cellular displacements and traction stresses exerted by cells on their ECM over time and during bacterial infection. The mechanical properties characterizing the domains are also shown. **(E)** Diagram illustrating the cell mechanotransduction mechanism that drives cell kinematics and dynamics in the cell computational model.

Our computational model relies on certain simplifications, namely, the cells are considered as linear elastic hexagonal prisms and are divided in three domains: the contractile, the adhesive and the expanding/protrusive (Figure 3A). We model cell-cell junctions as linear elastic elements in contact to each other (Figure 3B), and cell-ECM traction adhesions (i.e., focal areas at the ventral side of cells where traction stresses are exerted onto the underlying matrix) as surface-to-surface contacts (Figure 3C). The strength of the traction adhesions is characterized by the matrix parameter **K**, which links cellular traction stresses to cell and matrix displacements in all three different directions. Given our previous study, we also take into account that uninfected surrounding cells exert higher traction stresses as compared to nearby infected cells (Figure 3C). In the case of infection, for simplicity we assume that bacteria cannot replicate intracellularly or spread from cell to cell, so cells are either infected (red) or not (green) and their total number is fixed. In our *in silico* 3D monolayer the mechanical parameters of cells are based on previous experimental measurements (Bastounis et al., 2021b), and ECM stiffness is chosen so that it matches our experimental results and *in vitro* measurements (Figure 3D). The simulations were run using a finite element method (FEM) approach which allowed us to analyze the cell displacements and stresses in the whole considered geometry and according to the mechanotransduction mechanism depicted in Figure 3E.

### 3.3 *In silico* Model Predicts That Increased ECM Stiffness Promotes Mounding by Enhancing Cell Displacements and the Traction Stresses That are Generated by Nearby Uninfected Cells

Through our *in silico* model, we examined first whether cells on soft 3 kPa ECM as compared to stiff 35 kPa ECM would impart increased ECM displacements and exert reduced traction stresses *in silico* similar to what we previously determined *in vitro* (Figure 2). Not only were we able to validate our computational model but given the time efficiency of running *in silico* experiments, we sought to predict how would cells behave on ECM of intermediate stiffness (namely 10 and 20 kPa) and on stiff (∼2 GPa) glass typically used for *in vitro* experiments (Supplementary Figures S2A,B). We found that ECM displacements imparted by cells decreased following an exponential decay with increasing matrix stiffness. On the contrary, traction stresses increased monotonically reaching an asymptotic value at higher ECM stiffness.

**FIGURE 4:**
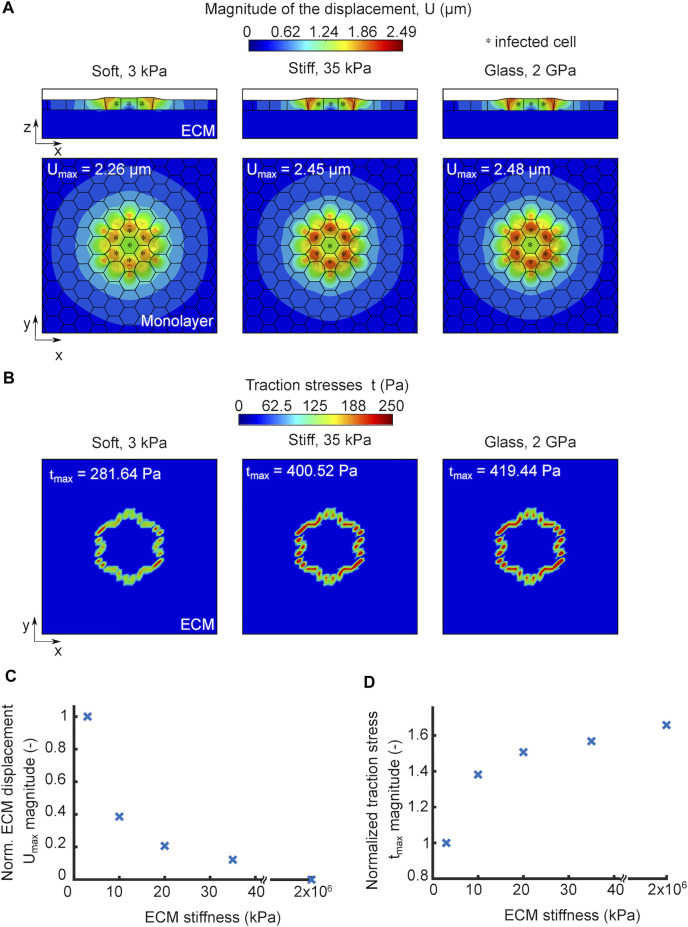
*In silico* model predicts that increased ECM stiffness promotes infected cell squeezing by enhancing cell displacements and traction stresses of nearby uninfected cells. **(A)** Orthogonal view maps of the magnitude of cellular displacements U (μm) of *in silico* infected cells residing on soft 3 kPa gel (left), stiff 35 kPa gel (middle) and 2 GPa glass substrate (right). Top (x–y) and side (x–z) views are shown in all three cases, and infected cells are denoted by an asterisk. An infection focus comprised by 7 cells is considered. The maximum cell displacement is indicated. **(B)** Orthogonal view maps of traction stresses exerted onto the ECM by the cells shown in panel A which have been exposed to *in silico* infection and reside on soft, stiff and glass matrices. The maximum traction stress is indicated. **(C)** Plot showing magnitude of maximum cell displacement (*U*_max_, *y*-axis) versus ECM stiffness (*x*-axis) for *in silico* cells in an infected monolayer. *U*_max_ values are normalized relative to *U*_max_ for cells residing on soft 3 kPa ECM. **(D)** Plot showing the magnitude of maximum traction stress (*t*_max_, *y*-axis) versus ECM stiffness (*x*-axis) for *in silico* cells in an infected monolayer. *t*_max_ values are normalized relative to *t*_max_ for cells residing on soft 3 kPa ECM. For C-D the infection focus consists of *N* = 7 infected cells.

We used our *in silico* model, to examine whether ECM stiffness would play a role in infection impacting the traction stress generating capacity of uninfected surrounder cells and, in turn, their mechanical competition with infected cells. We simulated infection by considering a cell monolayer residing on an ECM of a given stiffness and comprised by an infection focus containing just seven infected cells. We considered three different cases where the only parameter we varied was the ECM stiffness. Similar to our TFM experiments, we simulated infection in three different conditions, that is, with cells residing on 3 kPa ECM (soft), on 35 kPa ECM (stiff) or on a 2 GPa glass coverslip (glass), and let the simulations run for the same amount of time (one contraction-protrusion cycle). We noticed that cells in monolayers residing on a stiff matrix or glass exhibited larger cellular displacements compared to those residing on a soft matrix (Figure 4A). The maximum displacements observed in the simulations were 2.26, 2.45 and 2.48 μm for cells residing on soft matrix, stiff matrix and glass, respectively. Therefore, we can conclude that *in silico* cells within and near infection foci undergo larger displacements (directly related to squeezing of infected cells and subsequent mounding) when residing on stiff as opposed to softer ECM.

We previously showed that the ability of uninfected surrounders to produce strong traction stresses as they migrate towards the infected cells is key in squeezing the latter and eventually eliciting their extrusion. We thus wondered whether the more pronounced cellular displacements observed on stiff ECMs are driven by the increased traction stress generating capacity of uninfected, surrounding cells. To test that, we computed the traction stresses that cells exert on all three scenarios presented in Figure 4A and found the magnitude of traction stresses was higher for cells residing on stiffer matrices and predominantly high for surrounding, uninfected cells just at the edge of the focus since those are the ones that form protrusions in our model due to their tensional asymmetry (Figure 4B). The maximum traction stresses exerted by cells on the substrate were 281.64, 400.52 and 419.44 Pa for the soft, stiff and glass matrices, respectively. It is interesting that a 10-fold difference in ECM stiffness, when one compares cells residing on soft 3 kPa to stiff 35 kPa matrices, results in 42% increase in traction stresses, while a five order of magnitude increase in ECM stiffness, when comparing cells residing on 35 kPa hydrogels to 2 GPa glass, results in only 5% increase in the traction stress magnitude, suggesting that this mechanosensing mechanism is highly non-linear. Consistent with this, the change in maximum cellular displacements when comparing soft to stiff ECM is larger than when comparing stiff ECM to glass. When we ran simulations to examine the modulation of ECM displacements and traction stresses in a range of ECM stiffnesses for *in silico* cells during infection, we found the same trend as for non-infected cells (Supplementary Figures S2A,B and Figures 4C,D). The only difference between infected versus non-infected monolayers is that the extent to which traction forces increased with increasing ECM stiffness was larger during infection, likely because cells in this case are able to undergo protrusion due to development of stress asymmetries. Altogether, we conclude that increasing ECM stiffness gives rise to stronger cell-ECM traction stresses and enhanced cellular displacements, which is expected to increase infected cell squeezing and subsequent extrusion.

### 3.4 A Smaller Infection Focus or a Larger Difference in Stiffness of Uninfected Surrounders Relative to Infected Cells (Mounders) Increases the Squeezing of Infected Cells

**FIGURE 5:**
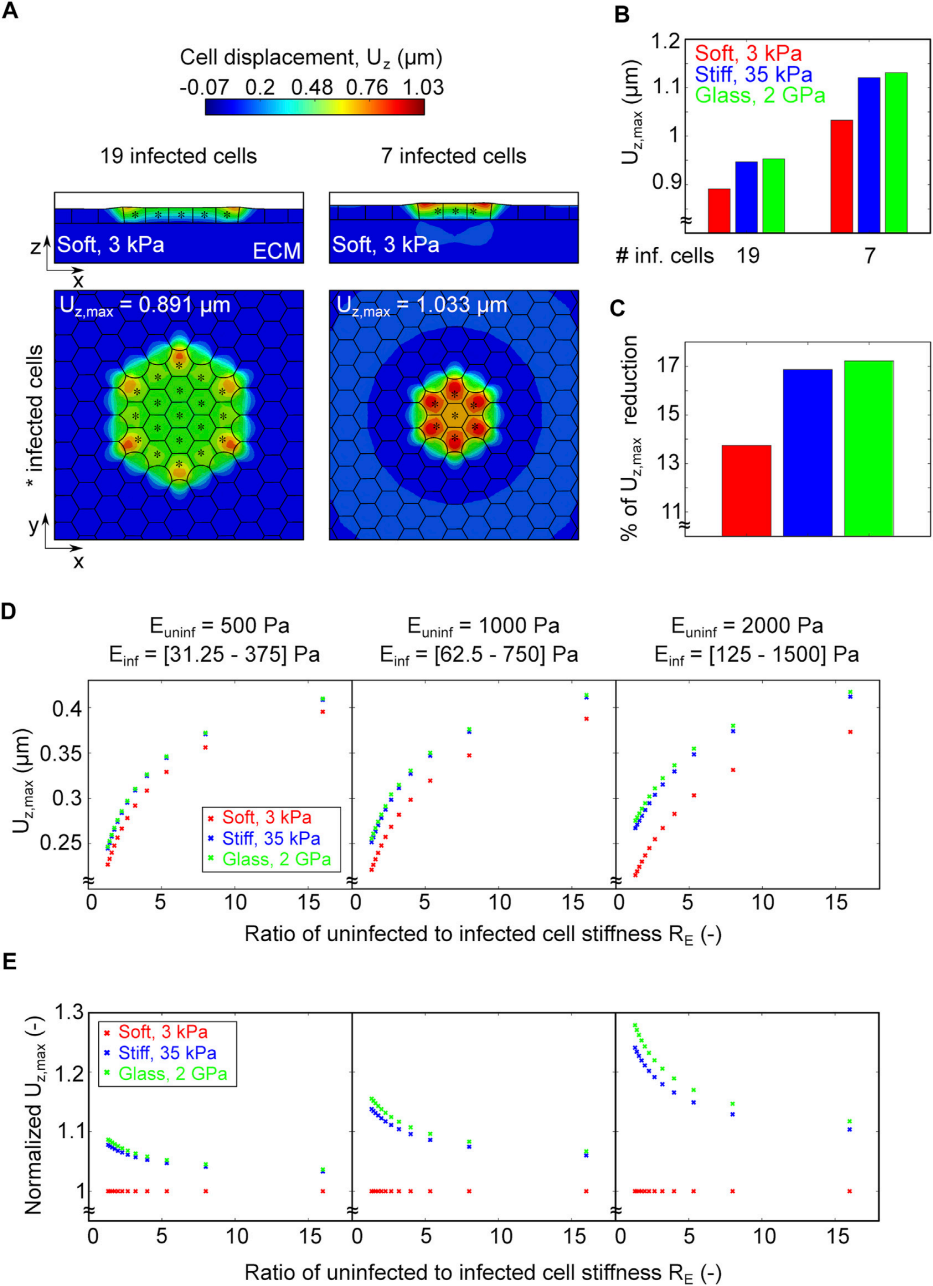
A smaller infection focus or a larger difference in the ratio of uninfected to infected cell stiffness increase infected cell squeezing. **(A)** Ortoghonal view maps of the magnitude of cellular displacements in the vertical axis U_*z*_ (μm) of *in silico* infected cells residing on a soft 3 kPa gel. On the left panel the focus is comprised by 19 infected cells denoted by asterisk, whereas on the right by 7. Top (x–y) and side (x–z) views are shown in both cases. **(B)** Barplot of the maximum vertical displacement U_*z*, max_ of cells residing on soft (red), stiff (blue) and glass (green) matrices in the case of a focus comprised by 19 or 7 infected cells. **(C)** Barplot of the relative decrease in U_*z*, max_ when comparing foci comprised of 7 relative to 19 infected cells for each of the varying stiffness matrices indicated in the panels above. **(D)** Plots of the maximum vertical displacement U_*z*, max_ (μm, vertical axis) versus the ratio of uninfected to infected cell stiffness (R_*E*_, horizontal axis) for *in silico* infection foci comprised of 7 infected cells and cells reside on soft 3 kPa (red), stiff 35 kPa (blue) and 2 GPa glass (green) matrix. In the simulations we assumed that the stiffness of the uninfected surrounder cells was fixed and equal to 500 Pa (left), or 1,000 Pa (middle), or 2,000 Pa (right), while infected cells could experience a range of stiffness shown below the plots (R_*E*_ = 1–16). **(E)** Same as panel D but *y*-axis shows U_*z*, max_ normalized to U_*z*, max_ exhibited by cells on a soft 3 kPa matrix.

Previous studies on endothelial cells infected with *L.m.* suggest that intercellular bacterial spread is enhanced when cells reside on softer ECM where enlarged infection foci are observed as compared to a stiffer ECM (Bastounis et al., 2018). To examine how the size of infection foci might impact cellular displacements and infected cell squeezing, we considered in our simulations two scenarios: 1) an infection focus comprised of just seven infected cells (small) and 2) a larger infection focus comprised of 19 infected cells (large). We also considered that these foci can be present on soft, stiff or glass ECM and run our simulations to compute the resulting cellular displacements (Figure 5A). We found that, independent of ECM stiffness, smaller infection foci result in cells undergoing larger cellular displacements and therefore, infected cell squeezing as compared to larger infection foci (Figure 5B). However, the percentage of reduction in maximum cell displacement when comparing small to large infection foci is more pronounced on stiffer as opposed to softer matrices, although the difference is subtle (Figure 5C).

An additional mechanical property that could change when host cells reside on soft versus stiff ECM is their passive stiffness arising from the organization of their cytoskeleton. Previous AFM measurements we conducted showed that, when residing on soft 3 kPa gels, infected cells at the edge of mounds exhibit a mean stiffness of 350 Pa while uninfected surrounding cells are 1,000 Pa stiff (Bastounis et al., 2021b). We also showed that changes in the ratio of uninfected to infected cell stiffness between the two populations are sufficient to lead to infected cell extrusion. Here we sought to determine whether and how changes in the ratio of uninfected to infected cell stiffness influence infected cell squeezing depending on ECM stiffness. To that end, we considered three distinct values of stiffness for uninfected surrounder cells, namely, 500, 1,000 and 2,000 Pa (left, center and right plot in Figure 5D). For each of these fixed values we ranged the stiffness of infected cells so that R_*E*_ (the ratio of uninfected to infected cell stiffness) ranges from 1 to 16. In addition, as in previous *in silico* experiments, we considered three different degrees of ECM stiffness, namely, soft, stiff and glass. We found that, irrespective of the stiffness of surrounder cells, the larger the value of R_*E*_, the higher the cellular displacements and thus infected cell squeezing. However, once R_*E*_ becomes higher than approximately 5, a slightly asymptotic behavior emerges, and the displacements stop increasing monotonically as if they had reached some plateau (Figure 5E). Moreover, we discovered that, irrespective of the stiffness of surrounder cells, for any given R_*E*_ the maximum cell displacements are lower for cells residing on soft matrices, while the displacements for cells residing on stiff matrix or glass are approximately the same, with those exhibited on glass being slightly higher than on stiff 35 kPa ECM. Interestingly, we found that the higher the absolute value of stiffness of uninfected surrounder cells, the stronger the difference in cellular displacements that cells undergo depending on ECM stiffness (Figures 5D,E). That is, for a given R_*E*_, if the stiffness of uninfected surrounders is larger (e.g., 2000 versus 500 Pa) then the effect of increased ECM stiffness in enhancing cellular displacements and therefore infected cell squeezing will be more prominent (Figure 5E). Altogether, these results suggest that a smaller infection focus and/or large differences in stiffness between surrounders and mounders, both promote large cell displacements and enhance infected cell squeezing. In addition, both these effects will be stronger if the ECM stiffness is increased, although for larger ECM stiffness the increase in cellular displacements stops being monotonic reaching asymptotic values which indicate a saturation in the cell mechanical sensitivity to its surrounding environment.

### 3.5 Monolayer Stresses are Concentrated at the Interface Between Infected and Surrounding Uninfected Cells Both Experimentally and in Our Computational Model

**FIGURE 6:**
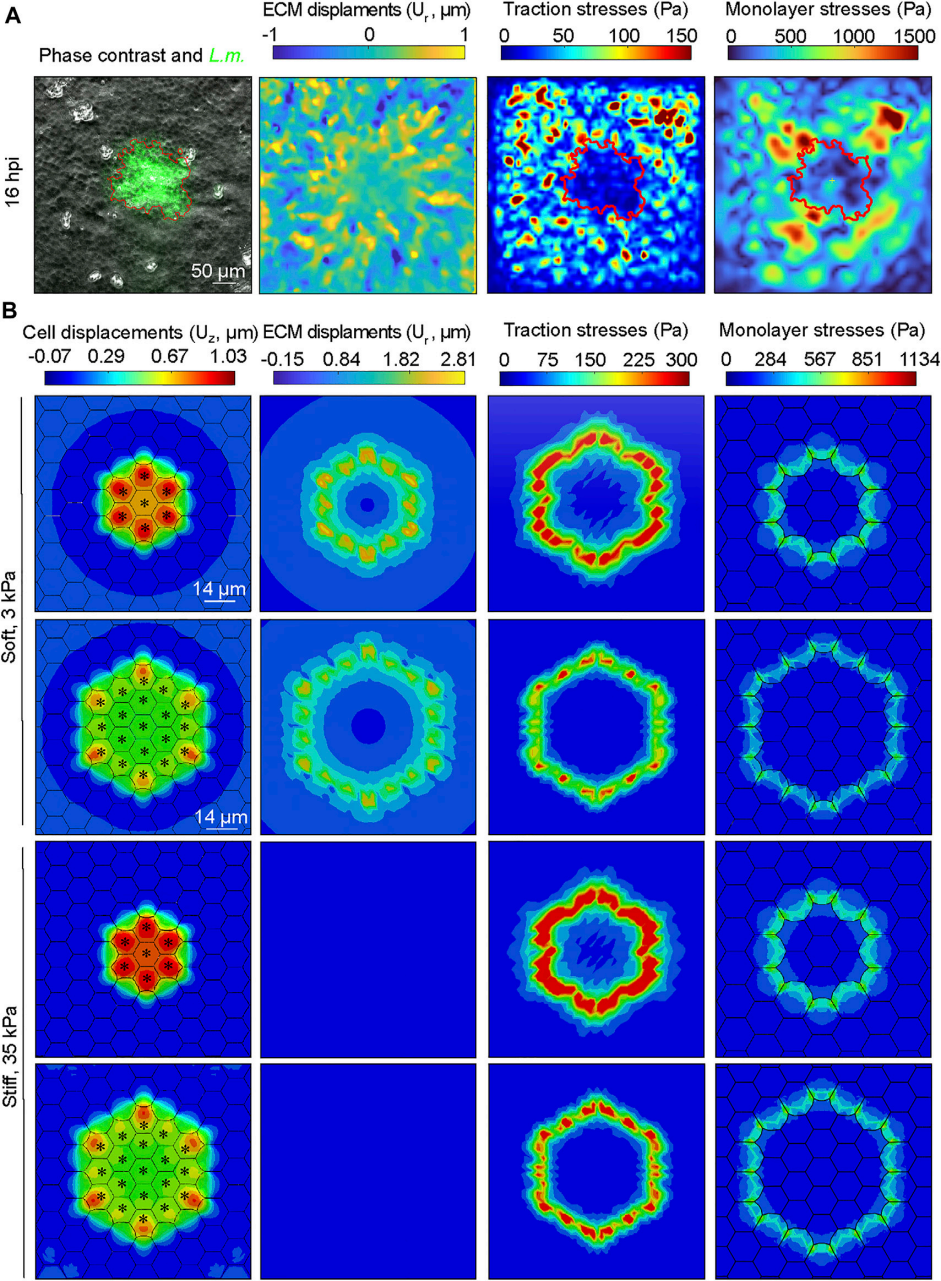
Monolayer stresses are concentrated at the interface between infected and surrounding uninfected cells both experimentally and in our computational model. **(A)** Exemplary image of MDCK cells residing on a soft 3 kPa hydrogel and infected with *L.m.* Time point shown refers to 16 h. p.i. Field of view imaged is centered around an infection focus. Columns show from left to right: phase contrast image overlayed with *L.m.* fluorescence (green), radial ECM displacements (*U*_*r*_: positive values indicate displacements pointing away from the focus center, μm), magnitude of traction stresses exerted by cells on the ECM (Pa), and magnitude of maximum monolayer tangential stresses (Pa). Red contour line indicates the area covered by infected cells (infection focus). **(B)** Same as panel A but the images depict the corresponding results of the infection computational simulations, with the exception of the first column which shows the configuration of the monolayer, the cell vertical displacements (μm) and where infected cells are denoted by an asterisk. First (second) row refers to a small (large) focus consisting of *N* = 7 (*N* = 19) infected cells residing on a soft 3 kPa ECM. Third (fourth) row refers to a small (large) focus consisting of *N* = 7 (*N* = 19) infected cells and cells reside on a stiff 35 kPa ECM.

Our simulations indicate that increasing the ratio of uninfected to infected cell stiffness between uninfected and infected cells enhances cellular displacements and squeezing of infected cells pertaining in the focus. This suggests that inter- and intra-cellular stresses at the interface between the two cell populations might play a critical role in promoting mounding. To test this, we first calculated the radial ECM displacements imparted by cells, the traction stresses they exert and the cell-cell stresses *in vitro* in an actual infection experiment focusing our attention on a single growing *L.m.* infection focus. We used TFM and Monolayer Stress Microscopy (MSM) to measure traction stresses as well as intra- and inter-cellular stresses (referred from here on as monolayer stresses) of cells residing on a soft 3 kPa hydrogel, prior to infected cell extrusion (Figure 6A and Supplementary Figure S3A). As previously shown, we noticed that the radial deformations *U*_*r*_ of surrounders just at the edge of the mound were large, indicative of them grabbing the ECM and pulling it away from the mound as they move directionally towards it. This traction stress orientation is not consistent with extrusion generated by a “purse-string” but is consistent with lamellipodial protrusion and directed cell migration (Kocgozlu et al., 2016). Thus, mounds are not caused by contraction of infected cells but rather by active crawling of uninfected surrounders that migrate toward the focus, squeezing and extruding the infected cells. Infected cells exerted reduced traction stresses compared to uninfected surrounders cells, consistent with previous findings (Bastounis et al., 2021b). Maximum monolayer tangential stresses were also lower for infected cells compared to uninfected surrounders (Figure 6A, fourth column). Interestingly, the maximum monolayer tangential stresses were localized at the edge of the infection focus, exactly where uninfected surrounder cells forcefully and actively moved towards the infection focus while pulling the ECM away from it, to eventually squeeze and extrude infected cells (Figure 6A, fourth column).

We then sought to examine whether similar behavior would be observed in our *in silico* model and used this opportunity as a means to validate our model but also to examine how monolayer stresses would be modulated *in silico* under conditions of varying ECM stiffness and focus size (Figure 6B and Supplementary Figure S3B). Consistent with the *in vitro* observations, radial ECM displacements were positive for the surrounder uninfected cells proximal to the infection focus and much larger for cells residing on soft as opposed to stiffer ECM, (Figure 6B, see second column). Moreover, both cellular traction stresses and monolayer tangential stresses exhibited the same tendency as in the *in vitro* experiment, including a high concentration of monolayer stresses at the interface of infected and non-infected cells (Figure 6B, see third and fourth columns). The range of values was within the same order of magnitude as that of the *in vitro* experiment, which validates our model despite the large variability in experimental observations. When inspecting the stress maps at the interface between infected and surrounding uninfected cells for cells residing on soft 3 kPa ECM, we found that both traction and monolayer stresses are increased to a higher extent around small as compared to large infection foci (*t*_max_ increases 13%, and *σ*_max_ increases 6%). We then wondered how stresses would be modulated at small as compared to large infection foci, for cells residing on stiff 35 kPa ECM. We discovered the same trend as when cells reside on soft ECM, with the exception that the relative differences in the magnitude of the maximum traction stress and monolayer stress were higher when comparing small versus large infection foci for cells residing on 35 kPa ECM (*t*_max_ increases 21%, and *σ*_max_ increases 8%). Altogether, these findings reveal that at least *in silico* both traction stresses and monolayer stresses are increased for uninfected surrounder cells proximal to the infection focus when the focus size is smaller as opposed to larger, and that this effect is stronger for cells residing on stiff 35 kPa as compared to soft 3 kPa ECM.

### 3.6 *In vitro* Experiments Validate *In silico* Predictions Showing That Infected Cell Extrusion is Enhanced When Epithelial Cells Reside on Stiff as Opposed to Softer Matrices

**FIGURE 7:**
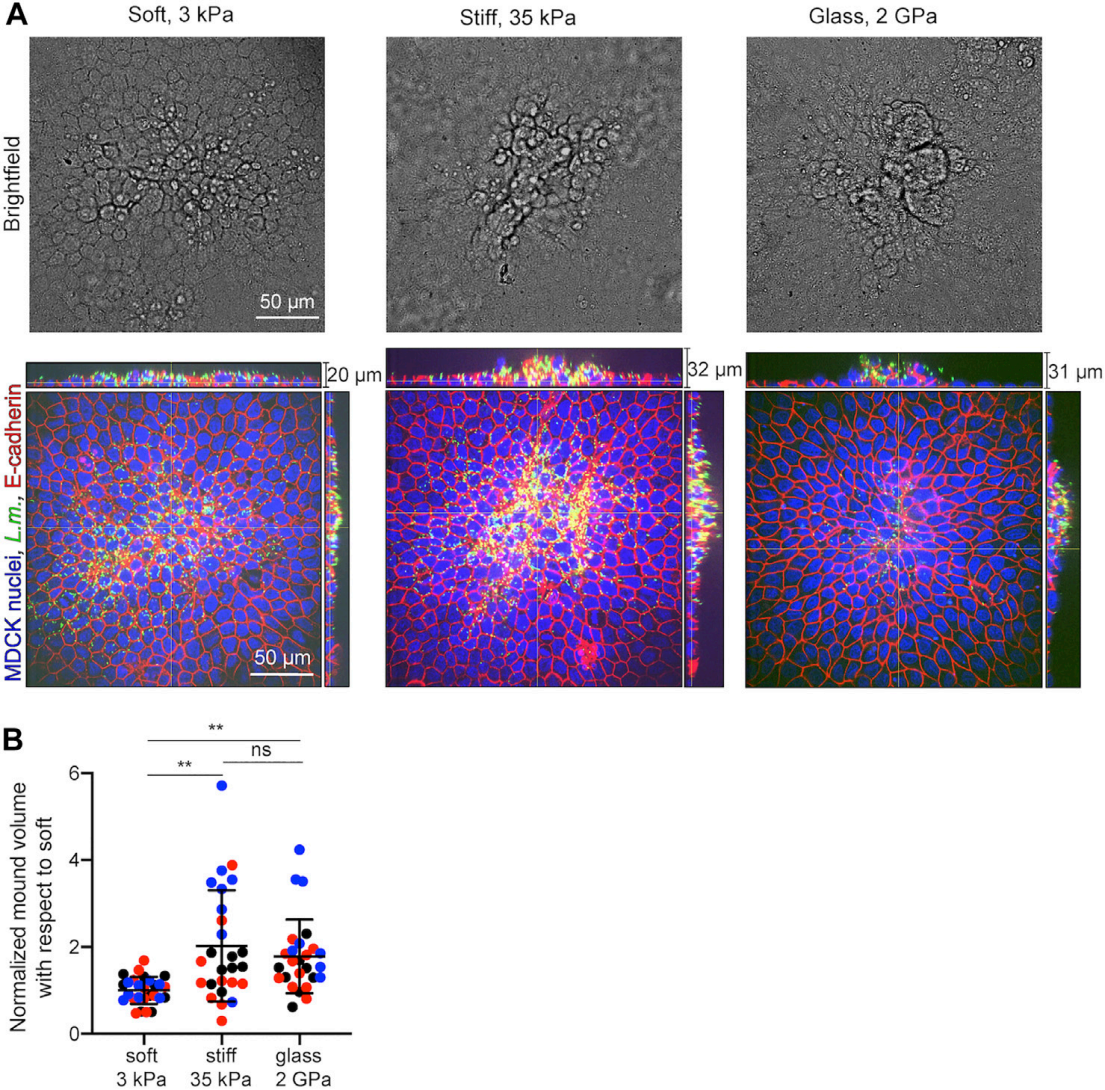
*In vitro* infection experiments validate the computational finding of increased infection mounding when cells reside on stiffer matrices. **(A)** Representative brightfield image (top row) and orthogonal views (bottom row) of *L.m.* fluorescence in green, E-cadherin in red and MDCK nuclei in blue for cells originated from a *L.m.*-infected well with the field of view shown focused around and infection focus at 24 hpi. MDCK cells resided on soft 3 kPa hydrogels (left), or stiff 35 kPa hydrogels (middle), or on glass coverslips (right). **(B)** Barplots of relative *L.m.*-infection mound volume at 24 hpi for MDCK cells residing on soft 3 kPa hydrogels, on stiff 35 kPa hydrogels, or on glass. For each of *N* = 3 independent experiments (shown in different color), values have been normalized relative to the mean mound volume of cells residing on a soft 3 kPa matrix and each circle corresponds to a distinct mound (N = 26 or 27 mound volumes quantified for each condition). (mean ± SD, Wilcoxon Rank Sum test: **: p < 0.01, ns: non-significant).

Our *in silico* infection experiments suggest that cells residing on stiffer matrices will undergo more prominent infected cell squeezing and subsequent extrusion as opposed to cells residing on softer matrices. To test whether the predictions of our computational model are correct, we seeded MDCK cells in monolayer on soft 3 kPa hydrogels, or stiff 35 kPa hydrogels, or on glass coverslips and infected them with low dosage of *L.m.* At 24 hpi samples were fixed and confocal microscopy imaging was performed to obtain z-stacks around infection foci. Although infected cell squeezing and extrusion were observed under all conditions, they were much more prominent for cells residing on stiff 35 kPa hydrogels or glass coverslips as opposed to soft 3 kPa hydrogels (Figures 7A,B). Moreover, especially on glass coverslips, surrounding uninfected cells appeared much more polarized as compared to the other two conditions (Figure 7A). When we quantified the volume of extruded cell domains relative to that which infected cells exhibit on a soft 3 kPa matrix using alpha shapes (Bastounis et al., 2021a), we found it to be 2-fold increased for cells residing on stiff 35 kPa matrices and 1.78-fold for cells residing on glass. Therefore, we can conclude that both computationally and experimentally a stiffer ECM promotes infection mounding and also that above a certain stiffness threshold an asymptotic behavior is reached and further increase in stiffness does not lead to further increase in mounding. Moreover, increased mounding observed for cells residing on stiffer ECM and glass was associated to decreased bacterial load (as evidence by the integral of the bacterial fluorescence) and decreased area of the infection focus (Supplementary Figure S4), reinforcing that infection mounding may limit bacterial spread across the basal cell monolayer as previously suggested (Bastounis et al., 2021b).

## 4 Discussion

The stiffness of the extracellular matrix (ECM) on which cells reside is a crucial determinant in modulating a variety of cellular functions such as cell motility, proliferation, and differentiation (Lo et al., 2000; Engler et al., 2006; LaValley et al., 2017). Here we showed that ECM stiffness also modulates the outcome of the cell competition that arises during intracellular bacterial infection of epithelial cells in monolayer and leads to the collective onslaught via extrusion of infected cells. Both our *in silico* model and the *in vitro* experiments show that increased ECM stiffness promotes the collective extrusion of infected cells as compared to a softer ECM. Through our *in silico* model, we found that the larger the difference in the ratio of stiffness between infected and uninfected surrounder cells, the larger will be the displacements that cells undergo and that lead to squeezing of infected cells and their subsequent extrusion. Moreover, through our computational model, we discovered that this effect is more prominent for cells residing on stiffer as opposed to softer ECM. Although *in vitro* experiments measuring cellular displacements, traction stresses or stiffness of infected versus non-infected cells and how those vary overtime as the infection focus grows on varying stiffness ECM were not conducted (we solely examined traction stress dynamics for infected cells at 3 kPa ECM *in vitro*), they could be the focus of future studies. Nevertheless, our *in vitro* measurements that reveal increased mounding for cells residing on stiff as opposed to softer ECM concern the computational predictions. Previous studies not involving infection have explored the role of physical cues in modulating the outcome of a mechanical cell competition (Matamoro-Vidal and Levayer, 2019; Gradeci et al., 2021). Consistent with our results, Gradeci et al. through a computational approach, showed that the higher stiffness of winner as opposed to loser cells during crowding leads to the compression of losers and an increase in their local density which is a prerequisite for their subsequent elimination (Gradeci et al., 2021). This study also showed that decreasing the ratio of stiffness of winner to loser cells will delay the kinetics of the ongoing competition rather than the final outcome. It is plausible to speculate that infection mounding could also occur for cells residing on soft matrices to the same extent as on stiff, if one was to inspect mounds at a later time point post-infection ( > 24 hpi). However, unlike the case of cell competition between wild-type and transformed cells where, only two cell populations are present, in the case of infection the landscape is more complex with some cells remaining uninfected, some being infected with few bacteria and others in the center of the focus being filled with bacteria. Moreover, it remains to be explored if delayed mounding means that bacteria can spread more at the basal cell monolayer (since cells are not extruded to the same extent) and thus, the number of infected cells still adhering to the substratum will increase faster than in the case when those are rapidly extruded, as occurs on a stiffer ECM.

Quite some studies have highlighted the importance of the relative difference in cell stiffness, adhesion strength, contractility and/or motility between two cell populations in regulating the outcome of a mechanical cell competition (Levayer et al., 2015; Matamoro-Vidal and Levayer, 2019). At the same time, it is well established that in many cell types, bulk cell stiffness and traction force generation tend to increase with increased ECM stiffness, as supported by our computational and experimental data for MDCK (Wells, 2008; Han et al., 2012). However, to our knowledge very few studies so far have explicitly tried to address the role of ECM stiffness in regulating any type of cell competition. Pothapragada et al. recently found that stiffening of the ECM attenuates extrusion of oncogenically transformed MDCK cells driven by their wild-type neighbors, which is the opposite of what we find in the case of infection (Pothapragada et al., 2022). In this study, however, which involves only two cell populations, cellular biomechanics were not characterized and the reasoning behind the enhanced elimination observed on soft ECM was the dynamic localization of the actin crosslinking protein filamin. On soft ECM filamin was found to localize at cell-cell contacts between transformed and non-transformed cells and that was necessary for driving the extrusion of the former. On stiffer ECM, instead, filamin localized perinuclearly. It is also worth noting that Pothapragada and colleagues documented a low number of extruded cells, which is distinct from the massive extrusion of infected cells we observe *in vitro*. It is likely that the mechanical mechanism driving extrusion in this case is different which could be due to the distinct biological process studied. Moreover, the authors of this study documented a bimodal extrusion pattern where extrusion counts were similar up to ECM stiffness of 11 kPa and then dropped to 50% less on ECMs 23 kPa or higher. On the contrary, we found significantly less infected cell extrusion when comparing soft 3 kPa ECM to stiff 35 kPa ECM, however the difference between stiff 35 kPa ECM and non-physiologically stiff glass coverslips is minimal, suggesting that, above a certain value, mechanical competition arising during infection becomes insensitive to stiffness and that saturation is reached above a stiffness around 35 kPa.

Our computational model suggests that ECM stiffness modulates the relative cell stiffness between infected and non-infected cells and that by itself it is sufficient to enhance infected cell squeezing on stiffer ECM. Moreover, the model predicts that a larger focus size will limit squeezing of infected cells as compared to a smaller focus size. These results raise the question of whether decreased infected cell squeezing observed on soft ECM is due to: 1) *L.m.* spreading more efficiently when cells reside on soft ECM; 2) the fact that the relative difference in stiffness of winners to losers is not as high as on a stiff ECM; or 3) both. Previous studies on endothelial cells showed that, when host cells resided on soft ECM, *L.m.* spread was favored, likely because of the decreased monolayer tension favoring the formation of bacterial protrusions and engulfment from the donor to the recipient cell (Bastounis et al., 2018). Thus, it is plausible that *L.m.* may also spread more efficiently between epithelial cells residing on soft as opposed to stiff ECM, leading to decreased ability of surrounders to squeeze infected cells since the infection focus becomes larger. This is consistent with the increased focus size we measure at 24 hpi. However, the increased relative stiffness difference between infected and non-infected cells could also play a role in promoting infected cell squeezing on stiffer ECM. Future studies could measure explicitly infected or not cell stiffness depending on ECM stiffness through atomic force microscopy (Buxboim et al., 2010; Bastounis et al., 2021b). Moreover, our computational model considers a fixed number of infected cells in each simulation, a simplification since *in vitro* the number of infected cells changes due to *L.m.* replicating and spreading intercellularly over time. Future studies could focus on incorporating the bacterial dynamics into the current computational model. Finally, it is worth mentioning that in our model we simulate one mechanotransduction cycle due to convergence issues arising while solving the relevant equations governing the dynamics of cell movement otherwise. *In vitro*, though, cells constantly sense and transmit forces from the microenvironment, thus going through multiple such cycles. Despite this limitation, the protrusion of uninfected surrounding cells is sufficient to allow us to inspect how infected cell squeezing and formation of mounds will be governed by the physical cues that cells display depending on their ECM stiffness. Finally, an additional limitation of the model is that the size and frequency of cell-cell and cell-ECM adhesions are considered constant regardless of the stiffness of the ECM on which cells reside. However, it was recently shown that cells can adapt their focal adhesion size and number based on ECM stiffness (Cao et al., 2017; Yeh et al., 2017). A more elaborate, future version of the computational model could account for changes in cell-cell and cell-ECM adhesion size as well as frequency. However, prior *in vitro* experiments will be necessary to investigate whether those change in a differential manner for uninfected surrounders versus infected cells.

Our discovery underscores the importance of ECM stiffness in regulating the cellular mechanical competition that arises during infection and leads to elimination of bacterially-infected cells. Intriguingly, in the small intestine, where food-borne infections like the one triggered by *L.m.* take place, ECM stiffness can increase significantly from typical values of few kPa in healthy conditions to several tenths of kPa in pathological states (*e.g.*, fibrosis) (Stewart et al., 2018; Onfroy-Roy et al., 2020). Studying further the dynamics of biochemical and (extra)-cellular physical signals during infection including using more elaborate computational models and live-cell biosensors will further reveal how those signals spatiotemporally crosstalk in health and during infection. This, in turn, will be critical to enhance our understanding of how healthy cells eliminate unfit ones during infection but also during other (patho)physiological processes involving cell competition.

## Data Availability

Data collected and computer codes are available on request to the corresponding authors, and also available for public download as indicated in the methods section. MSM codes can be downloaded here: https://github.com/ebastoun/Monolayer-Stress-Microscopy. The codes for calculating volume of extruded cell domains (mounds) can be downloaded here: https://github.com/ebastoun/Infection_mound_volume.
