# Supplementary material for: A Stiff Extracellular Matrix Favors the Mechanical Cell Competition that Leads to Extrusion of Bacterially-Infected Epithelial Cells
Source: Front Cell Dev Biol. 2022 Jun 22;10:912318. doi: 10.3389/fcell.2022.912318 (PMC9257086; doi:10.3389/fcell.2022.912318)
Supplement: Supplementary file 1 [file Presentation1.pdf]

frontiers

Supplementary Material

SUPPLEMENTARY MOVIE CAPTION

MOVIE S1 | Traction force microscopy on MDCK cells residing on soft or stiff matrices. Columns show: the phase contrast image of host MDCK cells (left), the cell-matrix deformations that cells impart onto their matrix (color indicates deformation magnitude in µm, middle) and the traction stresses (color indicates stress magnitude in Pa, right) exerted by the cells on their matrix. Scale bar and corresponding time are both indicated. Upper and lower rows refer to cells residing on a soft 3 kPa and a stiff 35 kPa hydrogel, respectively.

Suppl. Figure 1

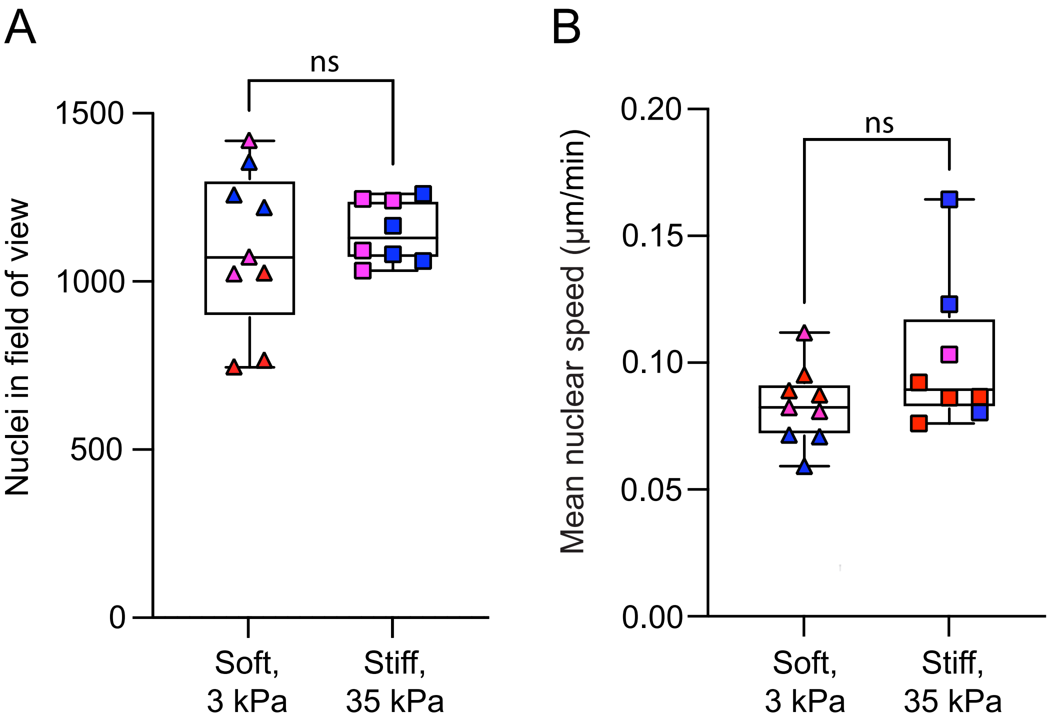

FIGURE S1 | Number of epithelial cell nuclei and migration speed is similar irrespective of ECM stiffness. **(A)** Boxplot of the number of nuclei in the whole field of view for the TFM recordings performed for MDCK cells residing on soft 3 kPa hydrogels (N=9 recordings) and stiff 35 kPa hydrogels (N=8 recordings). Same color points correspond to recordings performed the same day on different wells. Mean +/-SD is shown. Wilcoxon Rank Sum test reveals no significant difference (ns). **(B)** Boxplot of the mean speed of cell nuclei in the whole field of view for the TFM recordings performed for MDCK cells residing on soft 3 kPa hydrogels (N=9 recordings) and stiff 35 kPa hydrogels (N=8 recordings). Same color points correspond to recordings performed the same day on different wells. Mean +/- SD is shown. Wilcoxon Rank Sum test reveals no significant difference (ns). Related to Figure 2.

Suppl. Figure 2

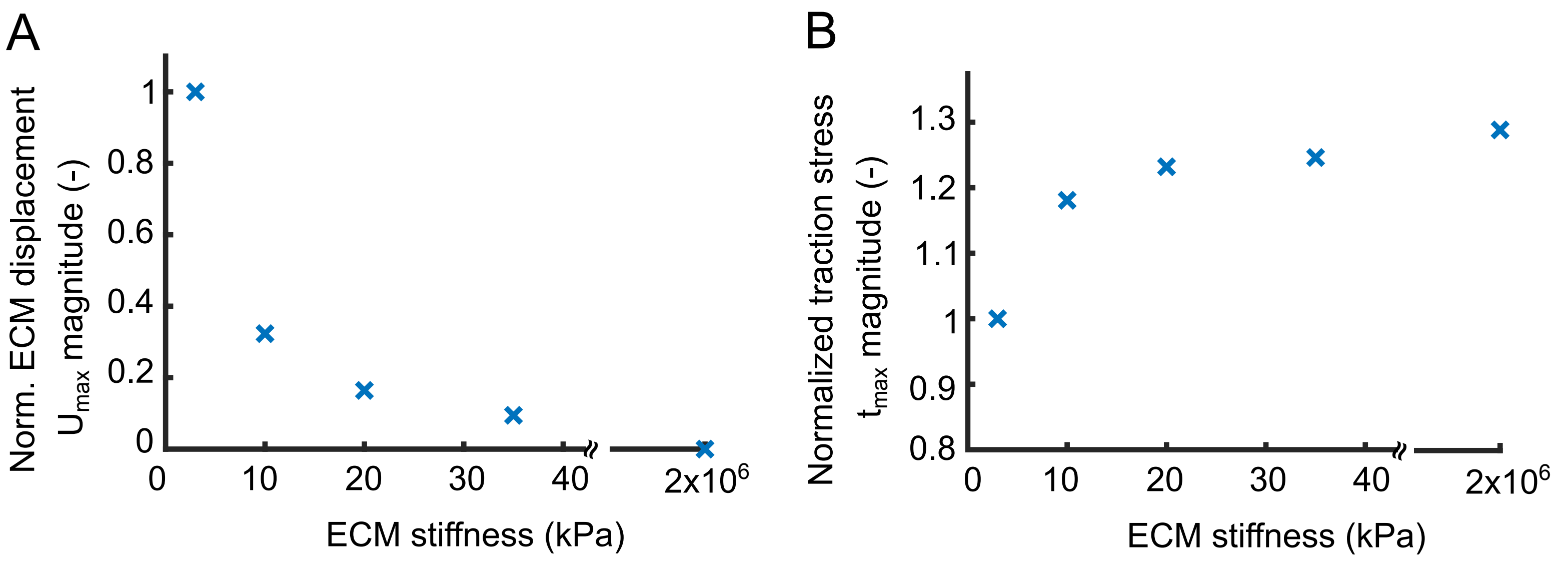

FIGURE S2 | *In silico* cells impart increased ECM displacements and exert reduced traction stresses when residing on softer as opposed to stiffer ECM. (A) Plot showing magnitude of maximum cell displacement (U_{max}, y-axis) versus ECM stiffness (x-axis) for *in silico* cells in monolayer in non-infected settings. U_{max} values are normalized relative to U_{max} for cells residing on soft 3 kPa ECM. **(B)** Plot showing magnitude of maximum traction stress (t_{max}, y-axis) versus ECM stiffness (x-axis) for *in silico* cells in monolayer in non-infected settings. t_{max} values are normalized relative to t_{max} for cells residing on soft 3 kPa ECM.

Suppl. Figure 3

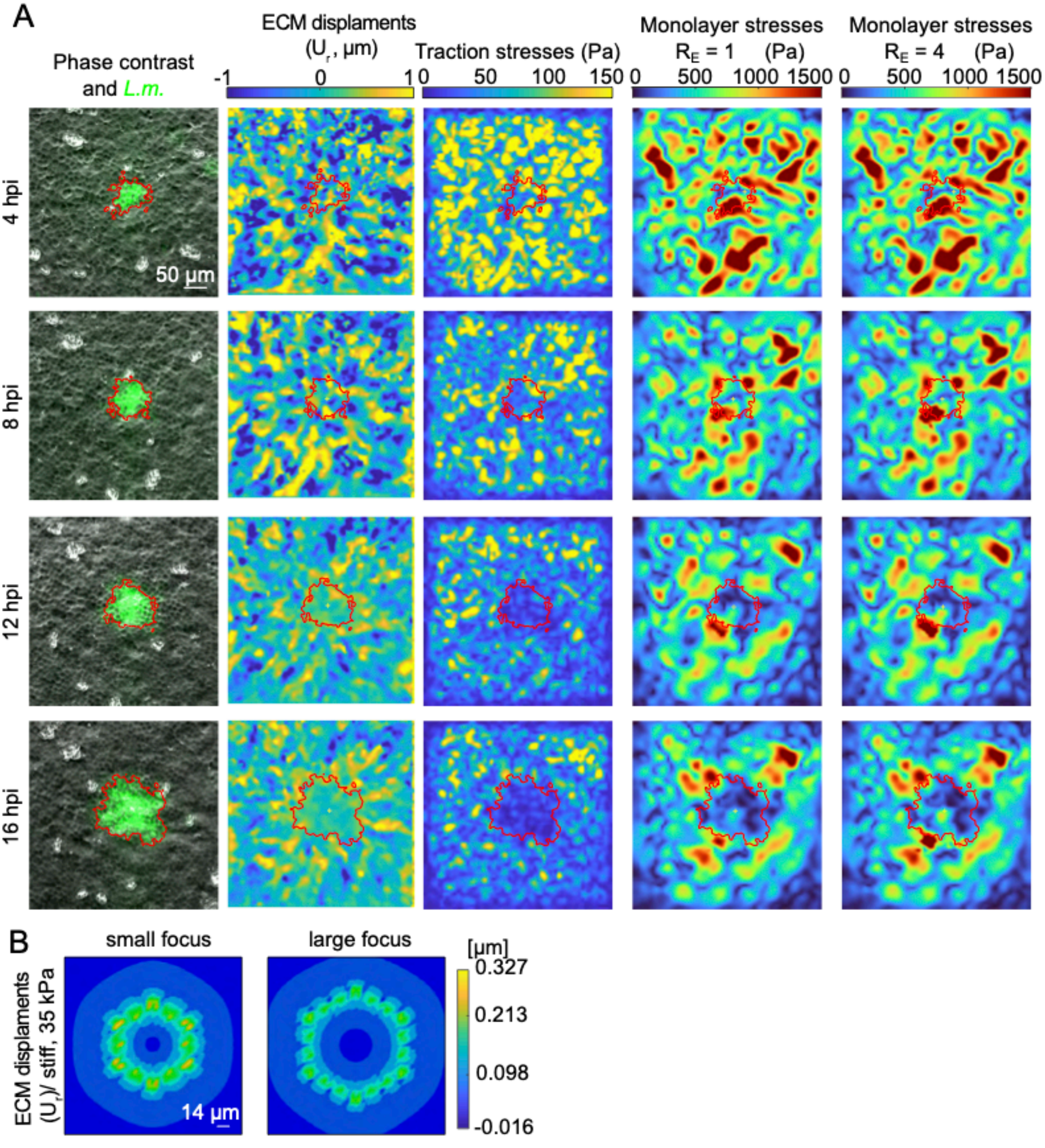

FIGURE S3| Monolayer stresses calculated through *in vitro* experiments accounting or not for the different stiffness between infected or not cells. (A) Exemplary images of MDCK residing on a soft 3 kPa ECM and infected with *L.m.* over 16 hpi (*i.e.*, prior to mounding initiation). Field of view imaged is centered around an infection focus. Columns show from left to right: phase contrast image overlayed with *L.m.* fluorescence (green), radial ECM displacements (U_r: positive values indicate displacements pointing away rom the focus center, µm), magnitude of cellular traction stresses, (Pa), maximum monolayer stresses (Pa) assuming $R_E = 1$, and $R_E = 4$. Red line indicates the infection focus area. **(B)** ECM displacements (U_r, µm) for cells residing on a stiff 35 kPa ECM for a small focus (N=7 infected cells, left) or a large focus (N=19 infected cells). Displacement maps are identical to those shown in Figure 6B in the third and fourth rows, but the colorbar has been changed so that the displacements' organization can be inspected.

Suppl. Figure 4

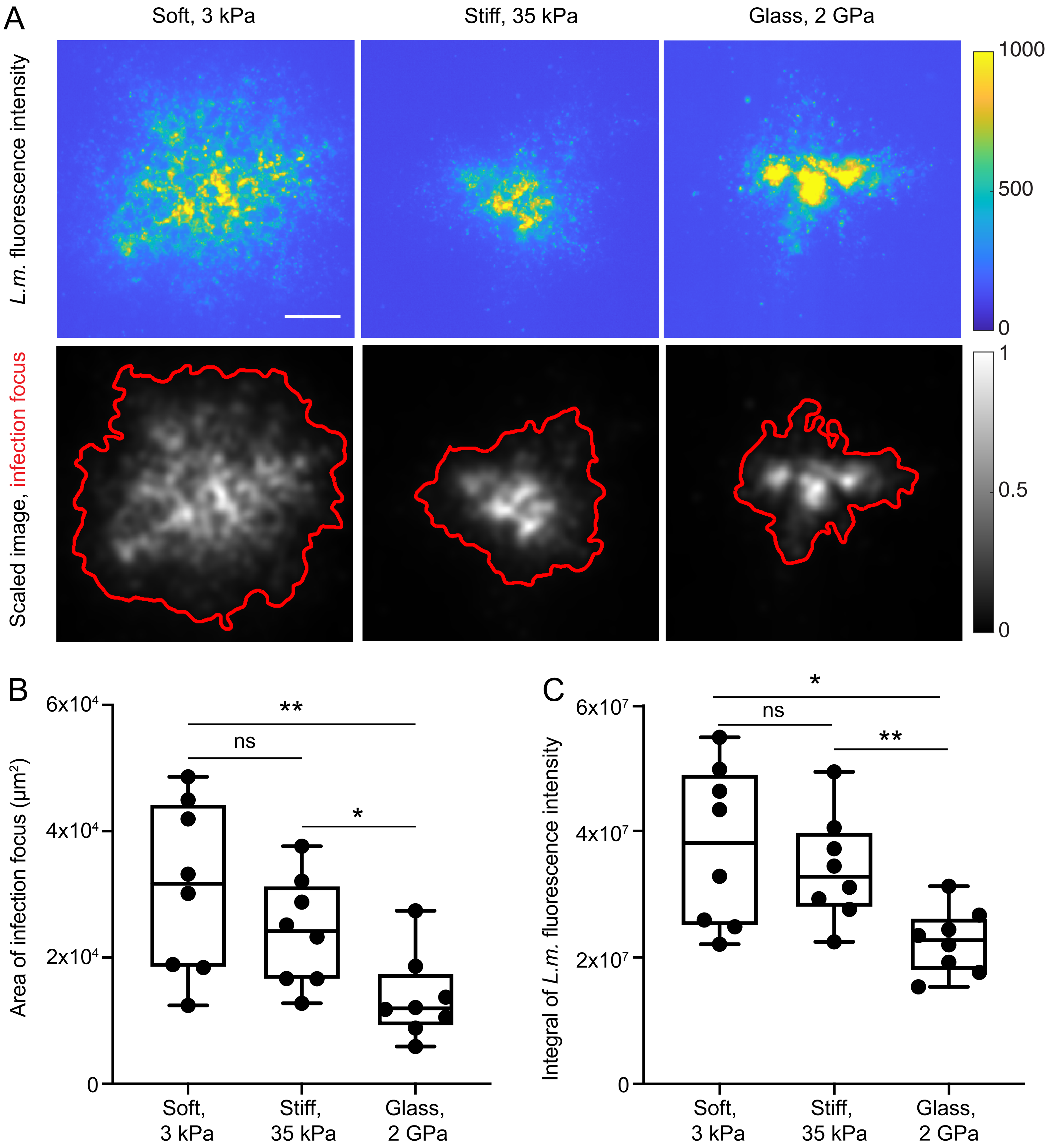

FIGURE S4 | Enhanced infection mounding observed in cells residing on stiffer matrices is accompanied by decreased bacterial spread through the basal cell monolayer. (A) Representative examples of infection foci for cells residing on soft 3 kPa hydrogels, stiff 35 kPa hydrogels or glass. First row shows the unprocessed image of the bacterial fluorescence. Second row shows the normalized image so that intensities range from 0 to 1 together with the contour of the infection focus (red). Scale bar 50 µm. **(B-C)** Boxplot of the area (µm2) of the infection focus (B) and of the integral of the bacterial fluorescence within the focus (C) on cells residing on soft 3kPa hydrogels, stiff 35 kPa hydrogels or glass. Wilcoxon Rank Sum test: ** $p<0.01$, * $p<0.05$, ns: non-significant. Related to Figure 7.
